# Checklist of the adventive psyllids (Hemiptera, Psylloidea) of the Hawaiian Islands, including new state records, identification key, and description of a new species of *Australopsylla*

**DOI:** 10.3897/zookeys.1279.178071

**Published:** 2026-05-05

**Authors:** Janis N. Matsunaga, Karl N. Magnacca, Paul D. Krushelnycky, Diana M. Percy

**Affiliations:** 1 Hawai‘i Department of Land and Natural Resources, Division of Forestry and Wildlife, 1151 Punchbowl St., Honolulu, Hawai‘i, 96813, USA University of Hawai‘i at Mānoa, Department of Plant and Environmental Protection Sciences Honolulu United States of America; 2 University of Hawai‘i at Mānoa, Department of Plant and Environmental Protection Sciences, 3050 Maile Way, Gilmore Hall 310, Honolulu, Hawai‘i, 96822, USA Hawai‘i Department of Land and Natural Resources, Division of Forestry and Wildlife Honolulu United States of America; 3 University of British Columbia, Botany Department and Biodiversity Research Centre, Vancouver, British Columbia, Canada University of British Columbia, Botany Department and Biodiversity Research Centre Vancouver Canada

**Keywords:** Dispersal, *
Eucalyptus
*, non-native, Spondyliaspidinae, Sternorrhyncha

## Abstract

In recent years, an increase in baseline arthropod surveys and closer scrutiny of specimens from current and past collections have resulted in a rise of adventive psyllid species discoveries in the Hawaiian Islands. In some cases, the new records reported here may have been known for some time but not officially published in the literature. In other cases, specimens remained unidentified for several years. We provide an updated checklist of the 23 adventive psyllid species in the Hawaiian Islands, including six new state records, and at least one new island record. The majority of recent psyllid establishment records, including those newly reported here, are in the subfamily Spondyliaspidinae (Aphalaridae). These records are overwhelmingly Australasian in origin, with host plant associations in the family Myrtaceae, predominantly *Eucalyptus*. One of these new Australasian-origin records, *Australopsylla exotica* Matsunaga & Percy, **sp. nov**., is described here as a new species from adults and immatures. A molecular systematic overview of the phylogenetic placement of native and adventive taxa includes an analysis of the position of *A. exotica* within *Australopsylla*.

## Introduction

The presence of adventive insects in many regions globally has increased more rapidly in recent years (Seebens et al. 2017), and the Hawaiian Islands are no exception (Mound et al. 2016; Hembry et al. 2021 and references therein). Moreover, models predict this trend will only increase (Seebens et al. 2021). Islands are considered particularly vulnerable to invasive species with detrimental impacts to both biological and economic aspects (Russell et al. 2017; Lenzner et al. 2020; Diagne et al. 2021). Land use, habitat degradation, and fragmentation, with increased trade and transport are considered the major drivers of adventive species spread (Lenzner et al. 2020). Small phytophagous sternorrhynchan insects (aphids, psyllids, scale insects, and whiteflies) are not capable of reaching the Hawaiian archipelago independently; they are either completely flightless or relatively weak fliers, and therefore abiotic and biotic dispersal agents are key (Antolínez et al. 2022). Many adventive psyllids worldwide are introduced with transported plant material during anthropogenic activities, particularly the horticultural trade in live plants (Beardsley 1991; Horton et al. 2021).

Recent surveys and checklists of psyllids and other insects in North America and Austro-Pacific regions have updated our knowledge of species diversity and considerably increased our understanding of adventive species spread (e.g., Mound and Wells 2015; Martoni et al. 2016, 2024, 2025; Mound et al. 2016; Martoni and Brown 2018; Halbert and Burckhardt 2020; Horton et al. 2021). The first comprehensive checklist of Hawaiian Psylloidea by Zimmerman (1948) has had subsequent updates by Nishida (2002) and Matsunaga et al. (2019). Here we provide an updated assessment of adventive psyllids in the Hawaiian Islands, including recent discoveries not previously known to occur in the Hawaiian Islands or not formally recorded in the literature. A combination of several newly established adventive psyllids, as well as a lack of clarity regarding some previous identifications and formal publication of older records, contributed to the necessity of this checklist. We provide an illustrated identification key to genera and species of adventive taxa, report new state and island records, and summarize the current and historical knowledge for each taxon. We describe a new species in the *Eucalyptus*-feeding genus *Australopsylla* Tuthill & Taylor recently found in the Hawaiian Islands and originating from Australia, and we provide clarification of the systematic and phylogenetic position of this taxon within *Australopsylla*. Additionally, we either provide new genetic resources or cite exemplars (e.g., publicly available DNA barcodes) for the adventive species and provide a phylogenetic overview of the systematic position of members of the Hawaiian psyllid fauna.

## Materials and methods

Recent discoveries of adventive psyllid species not previously known to occur in the Hawaiian Islands, or not formally recorded in the literature, were collected by KNM, PDK, and Hawai‘i Department of Agriculture and Biosecurity (**HDAB**) staff during baseline arthropod and targeted surveys for reported new pests. Tentative identifications were made by KNM, PDK, and JNM. Specimens of potential new records were checked against the Hawaiian Terrestrial Arthropod Checklist (Nishida 2002), Matsunaga et al. (2019), HDAB’s records and databases (both published and unpublished data), published literature, and collections at HDAB, the University of Hawai‘i Insect Museum, Honolulu, HI, USA (**UHIM**), Bernice Pauahi Bishop Museum, Honolulu, HI, USA (**BPBM**). To confirm final identifications, new records were reviewed by DMP and Cheryle O’Donnell (USDA, Systematic Entomology Laboratory, Washington, DC, USA, pers. comm.) and compared against the United States National Entomological Collection, Smithsonian Institution National Museum of Natural History, Washington, DC, USA (**USNM**) and DMP personal collection at the University of British Columbia, Vancouver, BC, Canada (**DMPC**). During this process, all known historic and recent records of native species were reviewed following the same methods to update the growing list of adventive Psylloidea. New state, island, and host record voucher specimens are deposited at the following museums and institutions: BPBM, DMPC, HDAB, UHIM, and USNM. Type material for *Australopsylla exotica* Matsunaga & Percy, sp. nov. is deposited in BPBM, with additional material in DMPC, HDAB, and USNM. Examination of related *Australopsylla* species was done from specimens in DMPC and images of paratype and other material at the Australian National Insect Collection, Canberra, Australia (ANIC). Our annotated checklist provides, at minimum, the following information for each adventive species: first detection/collection in the Hawaiian Islands, island distribution, host plants recorded from the Hawaiian Islands, plus brief summary of additional worldwide distribution and host records outside the Hawaiian Islands (not comprehensive), remarks, and genetic resources (not comprehensive). Family and subfamily classification follows Burckhardt et al. (2021). Table 1 provides an updated summary of all known psyllid species recorded from the Hawaiian Islands (adventive and native), the year first detected (for adventive species), island distribution, and known host plants in the archipelago.

**Table 1.** Summary of the Hawaiian psyllid fauna (96 species) showing adventive species with year first detected (Det) in bold; the ψ symbol indicates if considered formally published as a new state record here (includes some species known for some time but only previously recorded in unpublished or informal reports); island distribution (Ka: Kaua‘i, Oa: O‘ahu, Mo: Moloka‘i, Ln: Lāna‘i, Ma: Maui, Ha: Hawai‘i, an asterisk indicates uncertain establishment or could not be verified); host plant in HI (shown in [ ] if unconfirmed). For the adventive taxa, further details are given in the Remarks subsection for each taxon.

Family	Species	Det	Ka	Oa	Mo	Ln	Ma	Ha	Host plant in HI [unconfirmed]
Aphalaridae	***Aphalara rumicis* Mally, 1894**	**1947**		Oa*					[?]
	ψ ***Australopsylla exotica* Matsunaga & Percy, sp. nov**.	**2018**	Ka	Oa					*Eucalyptus camaldulensis*, *E. robusta*, *Eucalyptus* sp.
	***Blastopsylla occidentalis* Taylor, 1985**	**1993**	Ka	Oa		Ln	Ma*		*Eucalyptus camaldulensis*, *E. robusta*
	ψ ***Cryptoneossa triangula* Taylor, 1990**	**2018**						Ha	*Corymbia citriodora*
	***Ctenarytaina eucalypti* (Maskell, 1890)**	**1993**					Ma	Ha	*Eucalyptus globulus*, *Eucalyptus* sp.
	ψ ***Ctenarytaina longicauda* Taylor, 1987**	**2010**		Oa					*Lophostemon confertus*
	ψ ***Ctenarytaina spatulata* Taylor, 1997**	**2010**	Ka	Oa				Ha	*Eucalyptus camaldulensis*, *E. robusta*
	ψ ***Eucalyptolyma maideni* Froggatt, 1901**	**2018**		Oa				Ha*	*Corymbia citriodora*
	***Glycaspis brimblecombei* Moore, 1964**	**2001**		Oa			Ma	Ha	*Eucalyptus camaldulensis*, *E. robusta*, *E.* sp.
Calophyidae	***Calophya rubra* (Blanchard, 1852)**	**1989**					Ma		*Schinus molle*
Carsidaridae	***Macrohomotoma gladiata*Kuwayama, 1908**	**2022**		Oa					*Ficus microcarpa*
	***Mesohomotoma hibisci* (Froggatt, 1901)**	**2005**		Oa			Ma		*Hibiscus tiliaceus*
Liviidae	***Paurocephala cf. wilderi* Crawford, 1927**	**1925**			Mo*				[?*Ficus*]
Psyllidae	***Acizzia uncatoides* (Ferris & Klyver, 1932)**	**1994**	Ka	Oa	Mo	Ln	Ma	Ha	*Acacia* spp., *Albizia* spp.
	***Cacopsylla tobirae* (Miyatake, 1964)**	**2013**		Oa					*Pittosporum tobira*
	***Diaphorina citri*Kuwayama, 1908**	**2006**	Ka	Oa	Mo	Ln	Ma	Ha	*Citrus* spp., *Murraya paniculata*
	***Euceropsylla orizabensis* (Crawford, 1914)**	**2017**		Oa					*Pithecellobium dulce*
	***Heteropsylla cubana* Crawford, 1914**	**1984**	Ka	Oa	Mo	Ln	Ma	Ha	*Leucaena leucocephala*, *Samanea saman*
	***Heteropsylla fusca* Crawford, 1914**	**1986**		Oa					*Acacia* [=*Vachellia*] farnesiana
	***Heteropsylla huasachae* Crawford, 1914**	**1975**	Ka	Oa					*Acacia koa*, *Desmanthus virgatus*, *Samanea saman*
	ψ ***Heteropsylla texana* Crawford, 1914**	**1999**	Ka	Oa					*Neltuma pallida*
Triozidae	*Cerotrioza bridwelli* Crawford, 1920			Oa					*Xylosma hawaiiensis*
	*Cerotrioza bivittata* Crawford, 1918			Oa			Ma	Ha	*Xylosma hawaiiensis*
	*Crawforda triopsyllina* Caldwell, 1940				Mo	Ln			*Polyscias hawaiensis*
	*Hemischizocranium aloha* (Caldwell, 1940)		Ka						*Zanthoxylum dipetalum*
	*Hemischizocranium bessi* Tuthill, 1956							Ha	*Zanthoxylum dipetalum*
	***Heterotrioza chenopodii* (Reuter, 1876)**	**2010**		Oa					*Atriplex semibaccata*, *Chenopodium oahuense*
	*Hevaheva giffardi* Crawford, 1918							Ha	*Melicope* sp.
	*Hevaheva hyalina* Crawford, 1918							Ha	*Melicope* sp.
	*Hevaheva maculata* Caldwell, 1940		Ka						*Melicope* sp.
	*Hevaheva minuta* Crawford, 1928		Ka						*Melicope* sp.
	*Hevaheva monticola* Kirkaldy, 1908			Oa					*Melicope* sp.
	*Hevaheva perkinsi* Kirkaldy, 1902		Ka	Oa				Ha	*Melicope* sp.
	*Hevaheva silvestris* Kirkaldy, 1908		Ka	Oa					*Melicope* sp.
	*Hevaheva swezeyi* Crawford, 1928						Ma		*Melicope* sp.
	*Kuwayama lanaiensis* Uchida & Beardsley, 1992 [1992a]					Ln			*Pisonia sandwicensis*
	*Kuwayama minutura* (Caldwell, 1940)			Oa					*Pisonia sandwicensis*
	*Kuwayama oahuensis* Uchida & Beardsley, 1992 [1992a]			Oa					*Pisonia sandwicensis*
	*Kuwayama pisonia* Caldwell, 1940			Oa					*Pisonia sandwicensis*
	*Kuwayama tipicola* Caldwell, 1940			Oa			?Ma		*Sideroxylon polynesicum*
	*Kuwayama ventralis* Uchida & Beardsley, 1992 [1992a]		Ka						*Pisonia sandwicensis*
	***Leptynoptera sulfurea* Crawford, 1919**	**1977**	Ka	Oa	Mo		Ma	Ha	*Calophyllum inophyllum*
	*Megatrioza kauaiensis* Uchida & Beardsley, 1988		Ka						*Pritchardia* sp.
	*Megatrioza mauiensis* Uchida & Beardsley, 1988						Ma		*Pritchardia* sp.
	*Megatrioza molokaiensis* Uchida & Beardsley, 1988				Mo				*Pritchardia* sp.
	*Megatrioza palmicola* Crawford, 1918			Oa					*Pritchardia* sp.
	*Megatrioza zanthoxyli* Uchida & Beardsley, 1992 [1992b]							Ha	*Zanthoxylum hawaiiense*
	*Pariaconus caulicalix* Percy, 2017		Ka						*Metrosideros polymorpha*
	*Pariaconus crassiorcalix* Percy, 2017		Ka						*Metrosideros polymorpha*
	*Pariaconus dorsostriatus* Percy, 2017							Ha	*Metrosideros polymorpha*
	*Pariaconus elegans* Percy, 2017		Ka						*Metrosideros polymorpha*
	*Pariaconus gagneae* Percy, 2017		Ka						*Metrosideros polymorpha*
	*Pariaconus gibbosus* Percy, 2017						Ma		*Metrosideros polymorpha*
	*Pariaconus gracilis* (Crawford, 1918)			Oa	Mo		Ma		*Metrosideros polymorpha*
	*Pariaconus grandis* Percy, 2017		Ka						*Metrosideros polymorpha*
	*Pariaconus haumea* Percy, 2017		Ka						*Metrosideros polymorpha*
	*Pariaconus hawaiiensis* (Crawford, 1918)							Ha	*Metrosideros polymorpha*
	*Pariaconus hiiaka* Percy, 2017		Ka						*Metrosideros polymorpha*
	*Pariaconus hina* Percy, 2017						Ma		*Metrosideros polymorpha*
	*Pariaconus hualani* Percy, 2017				Mo				*Metrosideros polymorpha*
	*Pariaconus iolani* (Kirkaldy, 1902)		Ka						*Metrosideros polymorpha*
	*Pariaconus kapo* Percy, 2017							Ha	*Metrosideros polymorpha*
	*Pariaconus kupua* Percy, 2017						Ma		*Metrosideros polymorpha*
	*Pariaconus lanaiensis* (Crawford, 1918)				Mo	Ln			*Metrosideros polymorpha*
	*Pariaconus lehua* (Crawford, 1925)		Ka						*Metrosideros polymorpha*
	*Pariaconus liliha* Percy, 2017			Oa					*Metrosideros polymorpha*
	*Pariaconus lona* Percy, 2017				Mo				*Metrosideros polymorpha*
	*Pariaconus mauiensis* Percy, 2017						Ma		*Metrosideros polymorpha*
	*Pariaconus melanoneurus* Percy, 2017		Ka						*Metrosideros polymorpha*
	*Pariaconus minutus* (Crawford, 1918)							Ha	*Metrosideros polymorpha*
	*Pariaconus molokaiensis* (Crawford, 1927)				Mo				*Metrosideros polymorpha*
	*Pariaconus montgomeri* Percy, 2017						Ma		*Metrosideros polymorpha*
	*Pariaconus namaka* Percy, 2017			Oa					*Metrosideros polymorpha*
	*Pariaconus nigricapitus* (Crawford, 1918)				Mo	Ln		Ha	*Metrosideros polymorpha*
	*Pariaconus nigrilineatus* Percy, 2017							Ha	*Metrosideros polymorpha*
	*Pariaconus oahuensis* Percy, 2017			Oa					*Metrosideros polymorpha*
	*Pariaconus ohiacola* (Crawford, 1918)			Oa					*Metrosideros polymorpha*
	*Pariaconus pele* Percy, 2017							Ha	*Metrosideros polymorpha*
	*Pariaconus poliahu* Percy, 2017							Ha	*Metrosideros polymorpha*
	*Pariaconus proboscideus* Percy, 2017							Ha	*Metrosideros polymorpha*
	*Pariaconus pullatus* (Crawford, 1918)					Ln			*Metrosideros polymorpha*
	*Pariaconus pyramidalis* Percy, 2017							Ha	*Metrosideros polymorpha*
	*Pariaconus wyvernus* Percy, 2017							Ha	*Metrosideros polymorpha*
	*Paurotriozana adaptata* Caldwell, 1940			Oa					*Cryptocarya oahuensis*
	*Stevekenia aiea* Percy, 2017		Ka						*Nothocestrum peltatum*
	*Stevekenia nothocestri* Percy, 2017			Oa					*Nothocestrum longifolium*
	*Swezeyana atra* Percy, 2018			Oa					*Planchonella sandwicensis*
	*Swezeyana elongagena* Caldwell, 1940				Mo		Ma		*Planchonella sandwicensis*
	*Swezeyana hawaiiensis* Percy, 2018							Ha	*Planchonella sandwicensis*
	*Swezeyana magna* Percy, 2018		Ka						*Planchonella sandwicensis*
	*Swezeyana magnaccai* Percy, 2018			Oa					*Planchonella sandwicensis*
	*Swezeyana oahuensis* Percy, 2018			Oa					*Planchonella sandwicensis*
	*Swezeyana reticulata* Caldwell, 1940		Ka				?Ma		*Planchonella sandwicensis*
	*Swezeyana rubra* Percy, 2018							Ha	*Planchonella sandwicensis*
	*Swezeyana tentaculata* Percy, 2018		Ka						*Planchonella sandwicensis*
	*Trioza uniqua* (Caldwell, 1940)		Ka						*Cryptocarya mannii*

The illustrated identification key was made with reference to dry and slide-mounted specimens, and literature sources (Patch 1912; Ferris and Klyver 1932; Zimmerman 1948; Miyatake 1964; Mathur 1975; Hodkinson and White 1979; Yang 1984; Taylor 1985, 1990; Brown and Hodkinson 1988; Burckhardt 1988; Hollis and Broomfield 1989; Martin and Hollis 1992; Muddiman et al. 1992; Wheeler and Hoebeke 1997; Burckhardt et al. 1999; Mifsud and Burckhardt 2002; Hollis 2004; Mifsud and Porcelli 2012; Percy 2017a; Horton et al. 2018; Liao et al. 2019; Halbert and Burckhardt 2020; Percy et al. 2020; Martoni et al. 2025). Slide mounting methods follow those described previously (Percy 2017a). Briefly, ethanol-preserved material, and in some cases DNA vouchers, were cleared in 10% potassium hydroxide, followed by clove oil, and slide-mounted in Canada balsam as described in Hodkinson and White (1979). Specimens were imaged using a Zeiss Axioscope A1 microscope with Zeiss Axiocam 305 camera and imaging software ZEN v. 2.6; HeliconFocus v. 8.2.2 and Inkscape v. 1.2 were used to prepare images for publication; measurements were made with ImageJ (Rasband 1997–2018).

**Figure 1.**
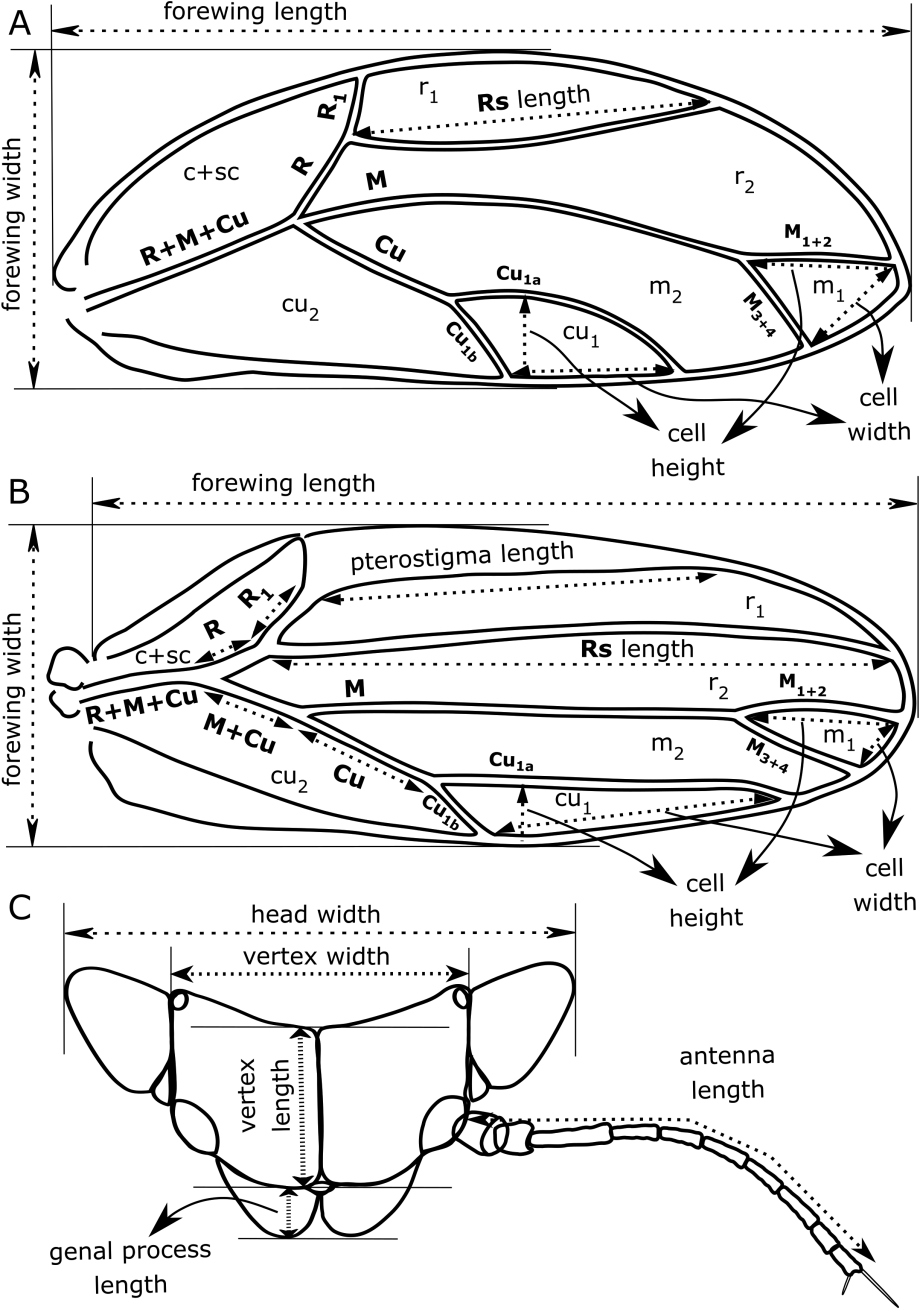
Guide to the type and placement of measurements referred to in the key: **A**. Triozid forewing (*Heterotrioza* type); **B**. Spondyliaspidine forewing (*Ctenarytaina* type); **C**. Head and antenna (*Ctenarytaina* type). Bold letters indicate forewing veins.

Abbreviations used in the identification key, description of *Australopsylla exotica* sp. nov. and Table 2 are as follows (all measurements are recorded in mm) and a guide to type and placement of forewing and head measurements is provided in Fig. 1. Adults: **WL**, forewing length; **WW**, forewing width; **HW**, head width; **AL**, antennal length; **PB**, distal proboscis segment length; **WL:WW**, ratio forewing length:width; **WL:RsL**, ratio forewing length:vein Rs length; **CUR**, ratio forewing cell cu_1_ width:height; **MR**, ratio forewing cell m_1_ width:height; **HW:VW**, ratio head width:vertex width; **VW:VL**, ratio vertex width:length; **VL:GC**, ratio vertex length:genal process length; **WL:HW**, ratio forewing length:head width; **AL:HW**, ratio antennal length:head width; **HW:HT**, ratio head width:hind tibia length; **HT:HF**, ratio hind tibia length:femur length. Adult male terminalia: **MP**, proctiger length; **PL**, paramere length; **AEL**, distal aedeagus segment length; **PL:HW**, ratio paramere length:head width; **MP:PL**, ratio proctiger length:paramere length; **PL:AEL**, ratio paramere length:distal aedeagus segment length; **PL:SH**, ratio paramere length:subgenital plate height. Adult female terminalia: **FP**, proctiger length; **FSP**, subgenital plate length; **RL**, anal ring length; **OVH**, ovipositor valvulae dorsalis height; **FP:RL**, ratio female proctiger:anal ring length; **FP:HW**, ratio female proctiger:head width; **FP:SP**, ratio female proctiger:subgenital plate length. Immatures: **BL**, body length; **BW**, body width; **WPL**, forewing pad length; **RW**, orifice (anal opening) width; **HW**, head width; **AL**, antennal length; **BL:BW**, ratio body length:width; **AL:HW**, ratio antennal length:head width. Eggs: **EL**, egg length; **EW**, egg width; **EL:EW**, ratio egg length:egg width.

**Table 2.** Comparative measurements (mm) and ratios of *Australopsylla* adults.

	*A. carinata* (1♀)	*A. exotica* (3♂, 6♀)	*A. revoluta* (2♂, 1♀)
Forewing length	3.63	2.25–3.03	3.09–4.17
Head width	0.97	0.74–0.88	0.95–1.08
Antenna length	1.15	0.98–1.16	1.52–1.54
Forewing length:width	2.56	2.43–2.63	2.71–2.84
Forewing length:pterostigma length	2.17	2.31–2.81	2.26–2.40
Forewing cell m_2_ width:height	0.32	0.42–0.52	0.38–0.44
Antenna length:head width	1.19	1.26–1.40	1.43–1.60
Female proctiger length:head width	0.63	0.58–0.64	0.60
Female proctiger length:anal ring length	4.07	3.25–3.43	3.61
Male paramere length:head width	–	0.24	0.33–0.35
Male proctiger length:paramere length	–	1.5	1.26–1.32

Genetic resources, if available, are listed for all but six species, and were either generated for this study (see Table 3) or exemplars obtained from public databases (e.g., GenBank). Where possible, we cite sequence data obtained from Hawaiian specimens. For data generated for this study, DNA was extracted from adults or immatures preserved in 70–95% ethanol. DNA extractions were performed using the Qiagen DNeasy Blood and Tissue Kit (Qiagen, Canada). Whole specimens were either punctured or partially bisected and placed in the lysis buffer with 10% Proteinase K to incubate overnight (12–15 h), after which specimens were removed and retained as vouchers (retained in DMPC unless otherwise noted). The remainder of the extraction followed the Qiagen protocol. Primers for the cytochrome oxidase I (COI) region were C1-J-1718: 5’-GGAGGATTTGGAAATTGATTAGTTCC-3’ (Simon et al. 1994) paired with H7005P-R: 5’-TGAGCTACTACRTARTATGTRTCATG-3’ (Percy and Cronk 2022), giving a length of 850 bp. Primers for the cytochrome B (cytB) region were cytBF: 5'-TGAGGNCAAATATCHTTYTGA-3' and cytBR: 5'-GCAAATARRAARTATCATTCDG-3' (Percy et al. 2018), giving a length of 385 bp. The PCR program was the same for both regions except the annealing temperature was 50 °C for COI and 56 °C for cytB (PCR program: denaturation at 94 °C for 3 min, then 40 cycles of 92 °C for 30 s, 50 °C (for COI) or 56 °C (for cytB) for 40 s, 72 °C for 1 min, with a final extension of 72 °C for 10 min). Genetic distances reported here were obtained using neighbour-joining analyses with uncorrected (p) distances in PAUP* (Swofford 2003). All sequences generated for this study (i.e., for *Australopsylla exotica* sp. nov., *Australopsylla* Tuthill & Taylor sp., *Blepharocosta* Taylor sp., *Ctenarytaina eucalypti* (Maskell), *C. longicauda* Taylor, *C. spatulata* Taylor, *Heteropsylla cubana* Crawford, *Phellopsylla* Taylor sp., and *Platyobria* Taylor spp.) have been submitted to GenBank and accession numbers provided in the genetic resources subsection for each taxon and Table 3. Complete mitochondrial genomes for the two previously described *Australopsylla* species (*A. carinata* (Froggatt) and *A. revoluta* (Froggatt)) were generated in a previous study (Percy et al. 2018), but for this study, the mitochondrial genomes have been annotated using Geneious R8 v. 8.1.9 (Biomatters Ltd., Kearse et al. 2012) and submitted to GenBank.

**Table 3.** Collection details and GenBank accession numbers for ten species sequenced (COI and cytB) for this study, plus in last two rows accession numbers for the annotated mitogenomes submitted to GenBank for this study. All DNA vouchers are deposited in DMPC.

Species	DNA voucher	Locality	COI	cytB
*Australopsylla exotica* Matsunaga & Percy, sp. nov.	KM125aOaAusFl21, KM125aOaAusFs21	USA, Hawaiian Islands, O‘ahu, Wai‘anae Mountains	PX507502–PX507503	PX527527–PX527528
*Australopsylla* sp. *sensu* Tuthill and Taylor (1955: 237)	AuAusSpF01	Australia, ACT, Canberra, near CSIRO	PX507504	PX527529
*Blepharocosta* Taylor, 1992 sp. [1992a]	AuBlephF02	Australia, NSW, Braidwood, north of Mongarlowe	PX507505	PX527530
*Ctenarytaina eucalypti* (Maskell, 1890)	Hi500MaCtenEucM14, Hi500MaCtenEucF14	USA, Hawaiian Islands, Maui, Makawao, Olinda Road	PX507506–PX507507	PX527531–PX527532
*Ctenarytaina longicauda* Taylor, 1987	DPHi76CtenspF14	USA, Hawaiian Islands, O‘ahu, northern Ko‘olau Mountains	PX507508	PX527533
*Ctenarytaina spatulata* Taylor, 1997	DPHi84CteneucF14	USA, Hawaiian Islands, O‘ahu, southern Ko‘olau Mountains	PX507509	PX527534
*Heteropsylla cubana* Crawford, 1914	DPHi36-13	USA, Hawaiian Islands, Hawai‘i south of Waimea	PX507510	PX527535
*Phellopsylla* Taylor, 1960 sp.	AuPhelloF02	Australia, NSW, Braidwood, north of Mongarlowe	PX507511	PX527536
*Platyobria* Taylor, 1987 sp. [1987c]	DPErioAu03	Australia, Tasmania	PX507512	PX527537
*Platyobria* Taylor, 1987 sp. [1987c]	TasPlatySpF03	Australia, Tasmania, ~4 km NE of Lilydale	PX507513	PX527538
Species	Percy et al. 2018 code	Locality	Annotated mitochondrial genome	
*Australopsylla carinata* (Froggatt, 1900)	DP1.idba.109	Australia, ACT, Namadgi	PX549493	
*Australopsylla revoluta* (Froggatt, 1900)	DP2.idba.142_circ	Australia, ACT, Tharwa	PX549492	

To provide a phylogenetic overview of the systematic placement of the adventive Hawaiian psyllid fauna, we took advantage of a published mitogenome phylogeny of the superfamily Psylloidea (Percy et al. 2018) to illustrate the phylogenetic position of all 23 adventive species relative to other species and the native genera. All but one (*Eucalyptolyma*) of the genera found in HI is represented in the molecular data, and genera are used to indicate placement of species where the species itself is not in the analysis. For taxa not included in the original mitogenome analysis of Percy et al. (2018), we employed a maximum likelihood (ML) backbone constraint analysis run with RAxML (v. 8.2.12) (Stamatakis 2014) on the CIPRES Science Gateway (Miller et al. 2010) to place the species in the mitogenome phylogeny based on the short COI and cytB sequences (for a more detailed description of this method, see Bastin et al. 2024). The constraint tree used was the total evidence tree obtained from mitogenome data presented in Percy et al. (2018).

## Results

To date, 23 adventive psyllids are recorded for the Hawaiian Islands, including six new state records (some of these known for some time but recorded in unpublished reports and now considered formally published here), plus at least one new island record (Table 1). The majority of recent psyllid establishment records, including those newly reported here, are in the subfamily Spondyliaspidinae (Aphalaridae). These records are overwhelmingly Australasian in origin, with host plant associations in the family Myrtaceae, predominantly *Eucalyptus*. Two adventive species, *Aphalara rumicis* Mally and *Paurocephala cf. wilderi* Crawford were recorded from a single individual in 1947 and 1925, respectively, with no establishment detected since.

### Key to the genera and species of adventive psyllids in the Hawaiian Islands

**Table:** 

1	Forewing with main stem vein R+M+Cu trifurcating (either strictly, e.g., Fig. 2A, B, or semistrictly, e.g., Fig. 2D, see dotted circles) into veins R, M, and Cu. Triozidae	**2**
–	Forewing with main stem vein R+M+Cu bifurcating into veins R and M+Cu (e.g., dotted circle in Fig. 2F, G)	**4**
2	Forewing uniformly wide almost entire length with margins more or less parallel and apex truncate giving a more or less rectangular shape, cell cu_1_ absent (Fig. 2A)	***Leptynoptera sulfurea* Crawford, 1919**
–	Forewing widest in the middle or apical third, with margins and/or apex curved to give a more oval appearance, cell cu_1_ present (Fig. 2B, D)	**3**
3	Forewing with apex acute, relatively low cell cu_1_ and narrow m_1_; aedeagus with a pair of apical processes (Fig. 2B, C)	***Heterotrioza chenopodii* (Reuter, 1876)**
–	Forewing mostly with apex rounded, if acute (e.g., *Cerotrioza*, *Kuwayama*, *Megatrioza*, *Swezeyana*) then vein Rs longer, or where wing apex acute and vein Rs of similar length (in *Pariaconus*) cell cu_1_ higher and cell m_1_ wider (Fig. 2D); aedeagus without apical processes (Fig. 2E)	**other Triozidae** (native species, see keys in Zimmerman 1948; Percy 2017a, 2017b, 2018)
4	Large species (WL > 3 mm). Carsidaridae	**5**
–	Smaller species (WL ≤ 3 mm)	**6**
5	Forewing extremely broad (WL:WW < 2.5), pterostigma extremely short and broad, usually with a distinct dark patch of pigmentation at the apex, and another dark area of variable size in cell cu_2_ along margin of vein Cu_1b_, without a false cross vein between veins M and Rs, cell cu_1_ extremely high (highest point above medial transverse line) (Fig. 2F); antennae short (subequal to head width)	***Macrohomotoma gladiata*Kuwayama, 1908**
–	Forewing narrower (WL:WW > 2.5), without pterostigma, and without areas of dark pigmentation (except for dark spots along the wing margin), with a false cross vein between veins M and Rs, cell cu_1_ relatively low (highest point below medial transverse line) (Fig. 2G); antennae long (> 2× head width)	***Mesohomotoma hibisci* (Froggatt, 1901)**
6	Male proctiger bipartite (e.g., Fig. 3E); hind coxa either with meracanthus absent or weakly developed, at most projecting as a blunt tubercle or papilla (e.g., Fig. 3Fa); associated with Myrtaceae hosts. Aphalaridae: Spondyliaspidinae	**7**
–	Male proctiger unipartite; hind coxa with well-developed thorn- or horn-shaped meracanthus (e.g., Fig. 3Fb); associated with other host plant families	**14**
7	Proximal segment of metatarsus with a single apical sclerotized spur (outer) (Fig. 3Ca); forewing as in Fig. 3A	***Blastopsylla occidentalis* Taylor, 1985**
–	Proximal segment of metatarsus with two apical sclerotized spurs (outer and inner) (e.g., Fig. 3Cb)	**8**
8	Forewing much wider in the apical half than basal half, vein Rs long and strongly curved to wing margin, length of vein M+Cu < 1/2 the length of vein Cu (Fig. 3G)	***Eucalyptolyma maideni* Froggatt, 1901**
–	Forewing more parallel sided, either slightly wider apically, or wider in the middle, vein Rs long but more or less straight or only slightly curving to wing margin, length of M+Cu > 1/2 the length of vein Cu (e.g., Fig. 3H)	**9**
9	Forewing apex acute or bluntly acute (Fig. 3I); antenna longer (AL:HW ≥ 2); genal processes as long or longer than vertex length (Fig. 3Jf)	***Glycaspis brimblecombei* Moore, 1964**
–	Forewing apex rounded (e.g., Fig. 3B, D, H); antenna shorter (AL:HW ≤ 1.5); genal processes shorter than vertex length (e.g., Fig. 3Jb–e)	**10**
10	Sexual dimorphism in forewing color, with females having a distinctly darker wing apex; forewing larger (WL > 2 mm), with cell cu_1_ elongate but relatively narrow (i.e., along wing margin) and extended towards wing base (CUR < 2), vein Cu_1a_ strongly arched and coming close to vein M, apex of vein Cu_1b_ curved towards wing margin (Fig. 7C, D)	***Australopsylla exotica* sp. nov**. (see also key to described *Australopsylla* species below and Table 3)
–	No sexual dimorphism in forewing color; forewing smaller (WL < 2 mm), with cell cu_1_ moderately to extremely low and wide (CUR ≥ 2), vein Cu_1a_ almost straight or weakly arched, vein Cu_1b_ more or less straight, apex not notably curved towards wing margin (Fig. 3B, H)	**11**
11	Male proctiger basal portion without digitate posterior projections, at most basal portion somewhat inflated posteriorly to an angled point (Fig. 3Eb–d); paramere more or less digitate, if bearing sclerotized pegs these irregular on posterior margin; female terminalia longer (FP ≥ HW) with anal ring ~ 0.3× proctiger length; mesotibia with small subapical longitudinal comb of densely-placed bristles (distinct from surrounding setae); forewing cell cu_1_ wide and low, vein Rs reaching margin prior to wing apex, vein R_1_ length to base of pterostigma approximately subequal to vein R length (Fig. 3B, D). *Ctenarytaina*	**12**
–	Male proctiger with basal portion bearing a pair of dorso-posterior projections at apex near intersection of basal and apical portions (Fig. 3Ea); paramere apical 2/3 broadly triangular with unbroken row of sclerotized pegs on anterior margin; female terminalia short (FP < HW) with anal ring ~ 0.5× proctiger length; mesotibia with short erect setae separated by at least their length but without subapical comb of dense bristles; forewing cell cu_1_ narrower, vein Rs reaching wing margin approximately at wing apex, vein R_1_ to base of pterostigma ~1/2 length of vein R (Fig. 3H)	***Cryptoneossa triangula* Taylor, 1990**
12	Generally larger species (WL ~1.5–2 mm) (Fig. 3B); male proctiger with apical portion much shorter, less than one quarter basal portion (Fig. 3Eb); paramere relatively short and narrowly digitate with length not exceeding bipartition of proctiger; female terminalia massive, proctiger dorsal surface not evenly concave and with a dark sclerotized hump immediately posterior of anal ring (Fig. 3Kb)	***Ctenarytaina longicauda* Taylor, 1987**
–	Generally smaller species (WL < 1–1.8 mm) (Fig. 3D); male proctiger apical portion longer, ~ 1/2 length or longer than basal portion (Fig. 3Ec, d); paramere relatively long and widening medially with length exceeding bipartition of proctiger; female terminalia smaller, proctiger with dorsal surface evenly concave, no sclerotized hump posterior of anal ring (Fig. 3Ka)	**13**
13	Slightly smaller species with head narrower relative to length (VW:VL ~ 1.5) and eyes in dorsal view more or less hemispherical (Fig. 3Jd); dorsal color of head and thorax typically darker brown to black; forewing with cells m_1_ and cu_1_ shorter (Fig. 3Da); male proctiger apical portion shorter than basal portion (Fig. 3Ec)	***Ctenarytaina eucalypti* (Maskell, 1890)**
–	Slightly larger species with head wider relative to length (VW:VL ~ 2.0) and eyes in dorsal view angular and elongate (Fig. 3Je); dorsal color of head and thorax typically lighter orange-brown; forewing with cells m_1_ and cu_1_ longer (Fig. 3Db), male proctiger apical portion longer, about as long as basal portion or slightly longer (Fig. 3Ed)	***Ctenarytaina spatulata* Taylor, 1997**
14	Forewing membrane clear or infused whitish-yellow or brown towards apex but without pigmented pattern of irregular spots, patches, or bands	**15**
–	Forewing membrane with pigmented pattern in irregular spots, patches, or bands (pattern may be faint in some individuals)	**21**
15	Proximal segment of metatarsus without apical sclerotized spurs; forewing vein M+Cu either extremely short, about one quarter length of vein Cu (Fig. 4A) or vein M+Cu longer than vein Cu (Fig. 4Ba)	**16**
–	Proximal segment of metatarsus with 2 apical sclerotized spurs (e.g., Fig. 3Cb); forewing vein M+Cu ~ 1/2 length of vein Cu (e.g., Fig. 4C–F)	**17**
16	Forewing widest in the middle third, apex narrowly rounded, vein M+Cu extremely short, about one quarter length of vein Cu, cell cu_1_ width approximately equal to height (Fig. 4A); metatibia without stout lateral sclerotized spurs along its length; head with genal processes long and slender (Fig. 4Gb); aedeagus as in Fig. 4Hb	***Calophya rubra* (Blanchard, 1852)**
–	Forewing widest in apical third, apex broadly rounded, vein M+Cu longer than vein Cu, cell cu_1_ width much greater than height (Fig. 4Ba); metatibia with 2 or 3 stout sclerotized spurs laterally along its length (similar in type to those apically) (Fig. 4Bb); head without genal processes	***Paurocephala cf. wilderi* Crawford, 1927**
17	Genal processes as long or longer than vertex length (Fig. 4Ga); aedeagus terminating in blunt hook (Fig. 4Ha)	***Cacopsylla tobirae* (Miyatake, 1964)**
–	Genal processes absent or if developed, shorter than vertex length (e.g., Fig. 4Gc); aedeagus terminating in acute or bluntly acute point (e.g., Figs 4Hc, 5B)	**18**
18	Forewing larger (WL > 2 mm), pterostigma small and relatively non-distinct, cells cu_1_ and m_1_ relatively high and narrow (Fig. 4D); antenna longer (AL:HW > 3); genal processes developed and conical (length slightly > 1/2 vertex length) (Fig. 4Gc)	***Euceropsylla orizabensis* (Crawford, 1914)**
–	Forewing smaller (WL < 2 mm), pterostigma broad and prominent, cells cu_1_ and m_1_ relatively low and wide (Fig. 4F); antenna shorter (AL:HW < 3, ~ ≤ 2); genal processes not developed (at most forming slight swellings). *Heteropsylla* in part (also refer to Halbert and Burckhardt 2020)	**19**
19	Paramere bipartite sections of subequal length with sclerotized apical tooth on posterior of outer section (Fig. 5A left); apex of distal aedeagus section more acute (Fig. 5Ba); female subgenital plate dorsal margin stepped with apex acute (Fig. 5Ca); forewing pterostigma typically longer (Fig. 4Fa)	***Heteropsylla cubana* Crawford, 1914**
–	Paramere bipartite sections of unequal length with inner section shorter and sclerotized apical tooth on anterior of inner section (Fig. 5Ab, c); apex of distal aedeagus section more blunt (Fig. 5Bb, c); female subgenital plate dorsal margin either slightly or not stepped with apex subacute to blunt (Fig. 5Cb, c); forewing pterostigma typically shorter (Fig. 4Fb, c)	**20**
20	Forewing membrane fuscous and cell m_1_ higher (Fig. 4Fb); bipartite sections of paramere deeply divided in posterior view (Fig. 5Ab); apex of distal aedeagus section not extended (Fig. 5Bb); female subgenital plate apex more or less rounded (Fig. 5Cb)	***Heteropsylla fusca* Crawford, 1914**
–	Forewing membrane clear and cell m_1_ lower (Fig. 4Fc); bipartite sections of paramere shallowly divided in posterior view (Fig. 5Ac); apex of distal aedeagus section extended (Fig. 5Bc); female subgenital plate apex subacute (Fig. 5Cc)	***Heteropsylla huasachae* Caldwell, 1941**
21	Antenna shorter than head width; forewing with Rs vein markedly sinuous towards apex (Fig. 6A)	***Diaphorina citri*Kuwayama, 1908**
–	Antenna longer than head width (barely so in *Aphalara*); forewing with Rs more or less straight or slightly sinuous (Figs 4E, 6B, 6C)	**22**
22	Forewing pterostigma absent (Fig. 6B); antenna short (AL:HW < 1.3); clypeus elongate, cylindrical, protruding ventrally and projecting anteriorly (Fig. 6Da)	***Aphalara rumicis* Mally, 1894**
–	Forewing pterostigma present (Figs 4E, 6C); antenna longer (AL:HW > 1.3); clypeus globular with base broadly rounded and not markedly protruding ventrally (Fig. 6Db)	**23**
23	Genal processes developed (short and conical, GC:VL ~0.5) (Fig. 6Ea); forewing membrane variably fuscous, from pale orange to dark orange-brown with extensive clouds of spots and bands of pigment mostly in the cells of the apical portion, forewing cells m_1_ and cu_1_ high (Fig. 6C); male proctiger greatly expanded basally and with digitate posterior projections (Fig. 6Fa)	***Acizzia uncatoides* (Ferris & Klyver, 1932)**
–	Genal processes not well developed (at most slight swellings) (Fig. 6Eb); forewing membrane clear with dark brown to black bands mostly bordering the veins towards the vein apices in the apical portion of the wing; forewing cells m_1_ and cu_1_ low (Fig. 4E); male proctiger only slightly expanded basally and without projections (Fig. 6Fb)	***Heteropsylla texana* Crawford, 1914**

**Figure 2.**
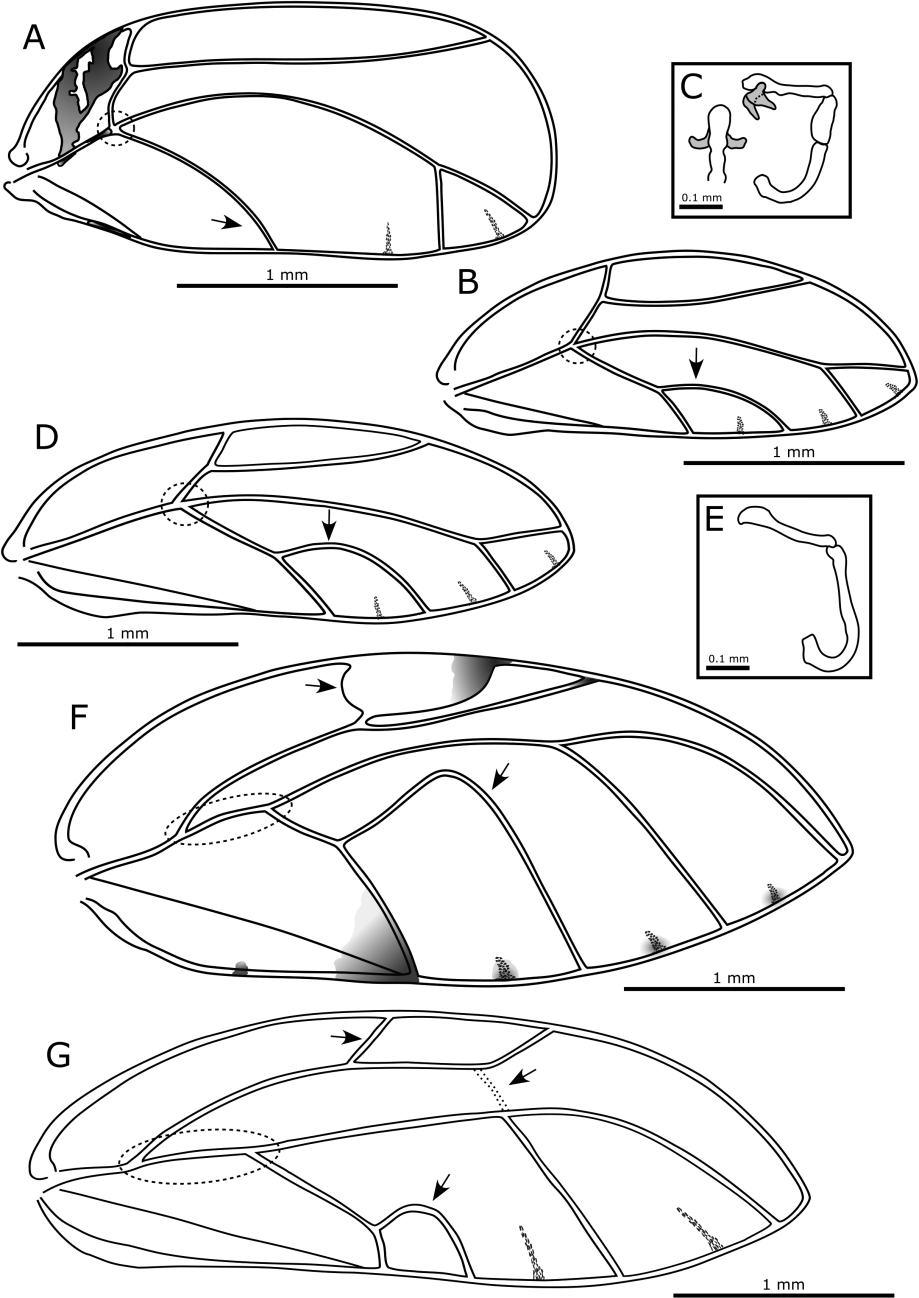
**A**. *Leptynoptera sulfurea* forewing; **B**. *Heterotrioza chenopodii*, forewing; **C**. *Heterotrioza chenopodii*, aeadeagus (lateral view, right; apical portion in dorsal view, left; apical processes in grey); **D**. *Pariaconus ohiacola*, forewing (this is the native species on O‘ahu with the most similar forewing structure to *Heterotrioza*); **E**. *Pariaconus ohiacola*, aedeagus (lateral view); **F**. *Macrohomotoma gladiata*, forewing; **G**. *Mesohomotoma hibisci*, forewing. Arrows and dotted ovals indicate characters referred to in the key.

**Figure 3.**
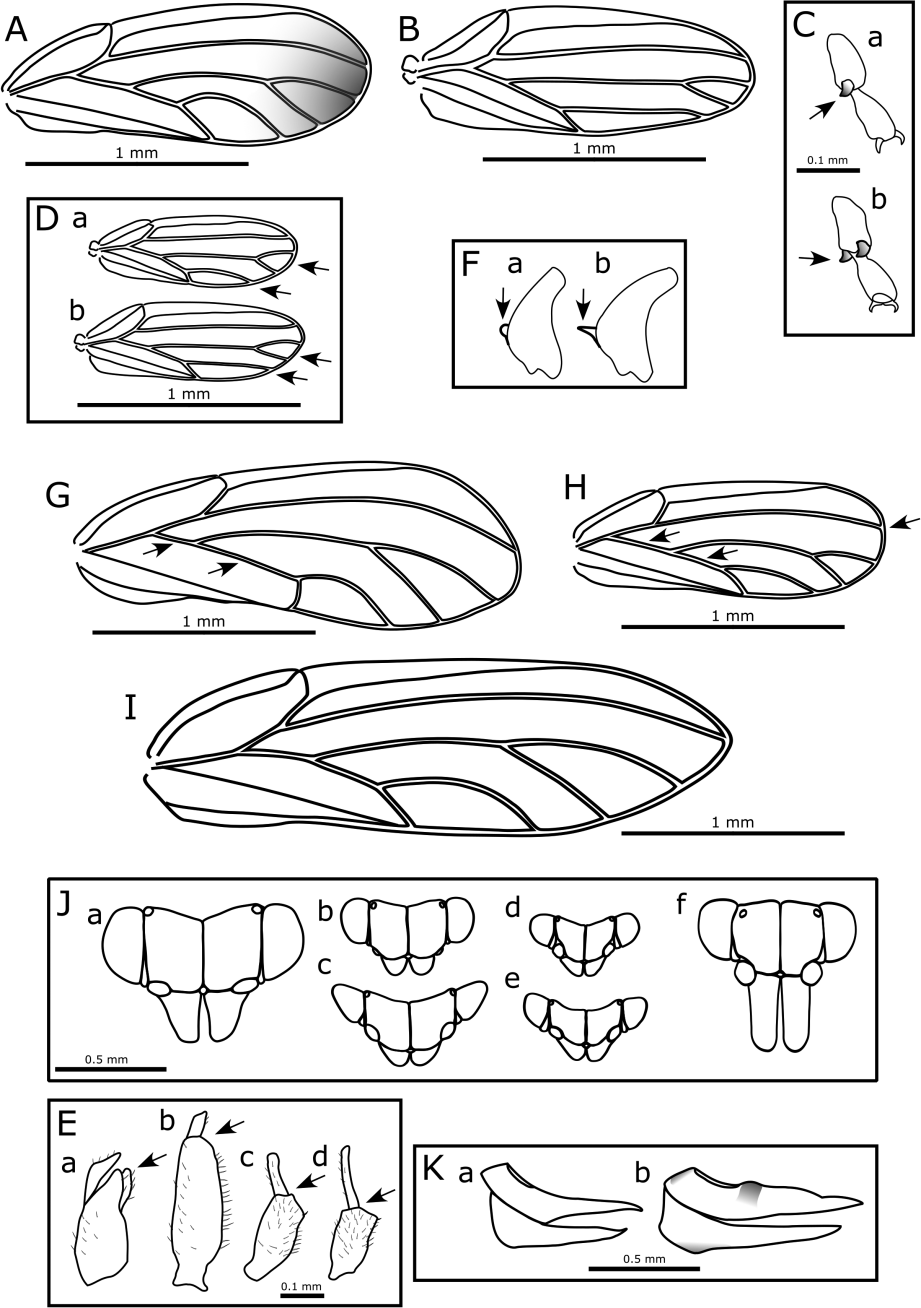
**A**. *Blastopsylla occidentalis*, forewing; **B**. *Ctenarytaina longicauda*, forewing; **C**. Examples of metatarsal spur number: **a**. One, **b**. Two; **D**. *Ctenarytaina* spp., forewings: **a**. *C. eucalypti*, **b**. *C. spatulata*; **E**. Male proctigers: **a**. *Cryptoneossa triangula*, **b**. *Ctenarytaina longicauda*, **c**. *C. eucalypti*, **d**. *C. spatulata*; **F**. Examples of meracanthi: **a**. *Ctenarytaina*, **b**. *Heteropsylla*; **G**. *Eucalyptolyma maideni*, forewing; **H**. *Cryptoneossa triangula*, forewing; **I**. *Glycaspis brimblecombei*, forewing; **J**. Head in dorsal view: **a**. *Eucalyptolyma maideni*, **b**. *Cryptoneossa triangula*, **c**. *Ctenarytaina longicauda*, **d**. *C. eucalypti*, **e**. *C. spatulata*, **f**. *Glycaspis brimblecombei*; **K**. Female terminalia: **a**. *Ctenarytaina eucalypti*, **b**. *C. longicauda*. Arrows indicate characters referred to in the key.

**Figure 4.**
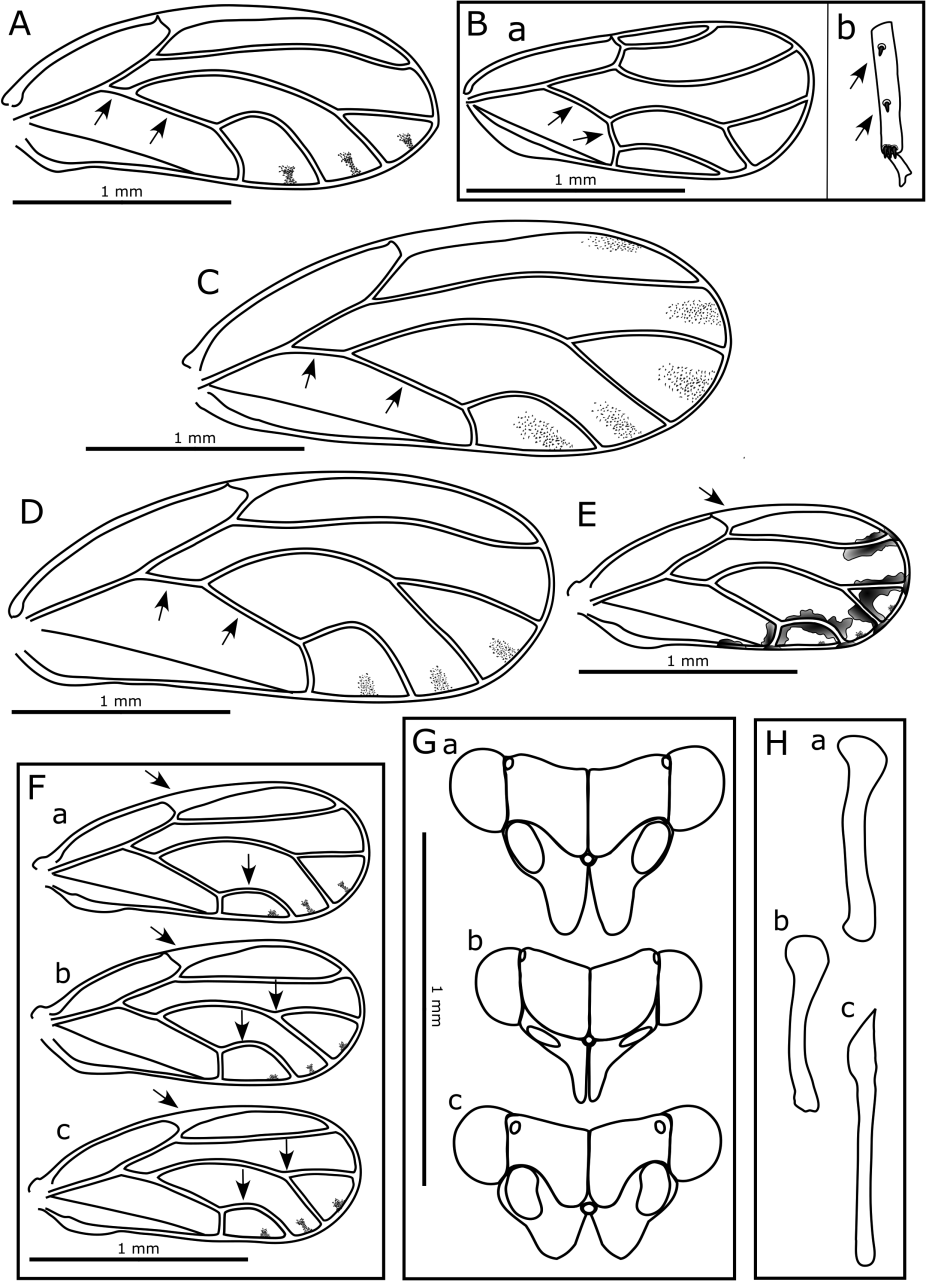
**A**. *Calophya rubra*, forewing; **B**. *Paurocephala cf. wilderi*: **a**. Forewing, **b**. Apical portion of metatibia showing lateral spurs; **C**. *Cacopsylla tobirae*, forewing; **D**. *Euceropsylla orizabensis*, forewing; **E**. *Heteropsylla texana*, forewing; **F**. Forewings: **a**. *Heteropsylla cubana*, **b**. *H. fusca*, **c**. *H. huasachae*; **G**. Heads in dorsal view: **a**. *Cacopsylla tobirae*, **b**. *Calophya rubra*, **c**. *Euceropsylla orizabensis*; **H**. Apical aedeagus segments: **a**. *Cacopsylla tobirae*, **b**. *Calophya rubra*, **c**. *Euceropsylla orizabensis*. Arrows indicate characters referred to in the key.

**Figure 5.**
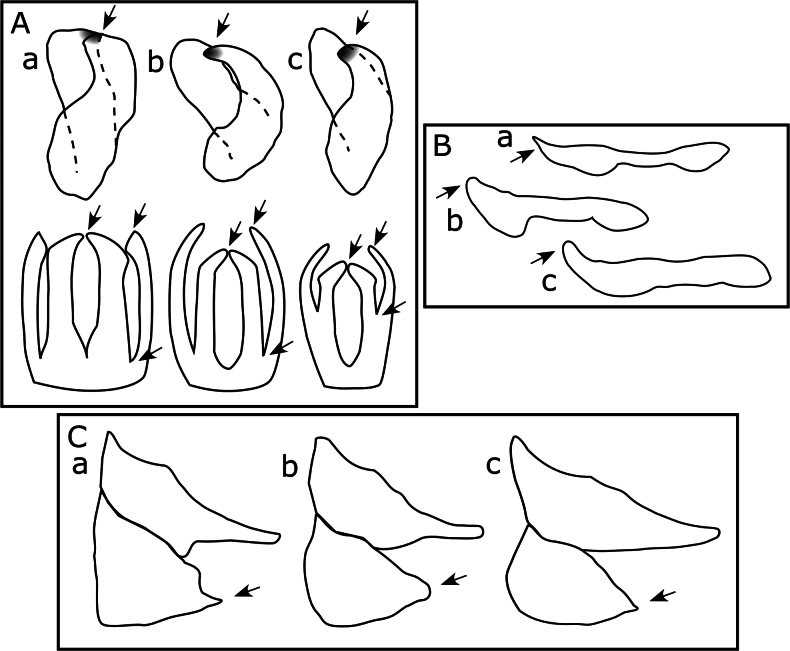
*Heteropsylla* spp. **A**. Parameres (above interior view, below posterior view): **a**. *H. cubana*, **b**. *H. fusca*, **c**. *H. huasachae*; **B**. Aedeagi: **a**. *H. cubana*, **b**. *H. fusca*, **c**. *H. huasachae*; **C**. Female terminalia: **a**. *H. cubana*, **b**. *H. fusca*, **c**. *H. huasachae*. Arrows indicate characters referred to in the key.

**Figure 6.**
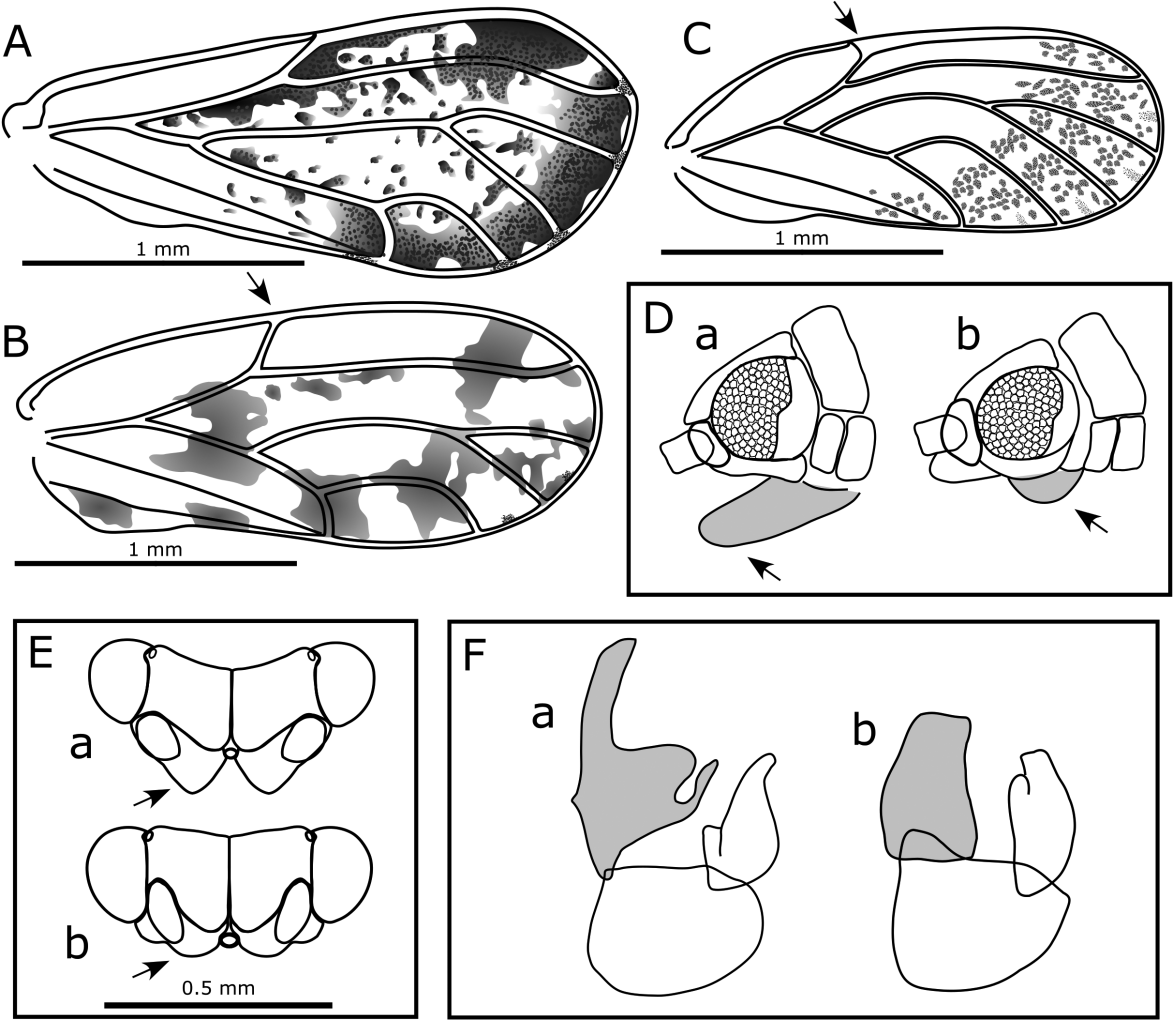
**A**. *Diaphorina citri*, forewing; **B**. *Aphalara rumicis*, forewing; **C**. *Acizzia uncatoides*, forewing; **D**. Heads in lateral view (clypeus in grey): **a**. *Aphalara rumicis*, **b**. *Acizzia uncatoides*; **E**. Heads in dorsal view: **a**. *Acizzia uncatoides*, **b**. *Heteropsylla texana*; **F**. Male terminalia (proctiger in grey): **a**. *Acizzia uncatoides*, **b**. *Heteropsylla texana*. Arrows indicate characters referred to in the key.

**Figure 7.**
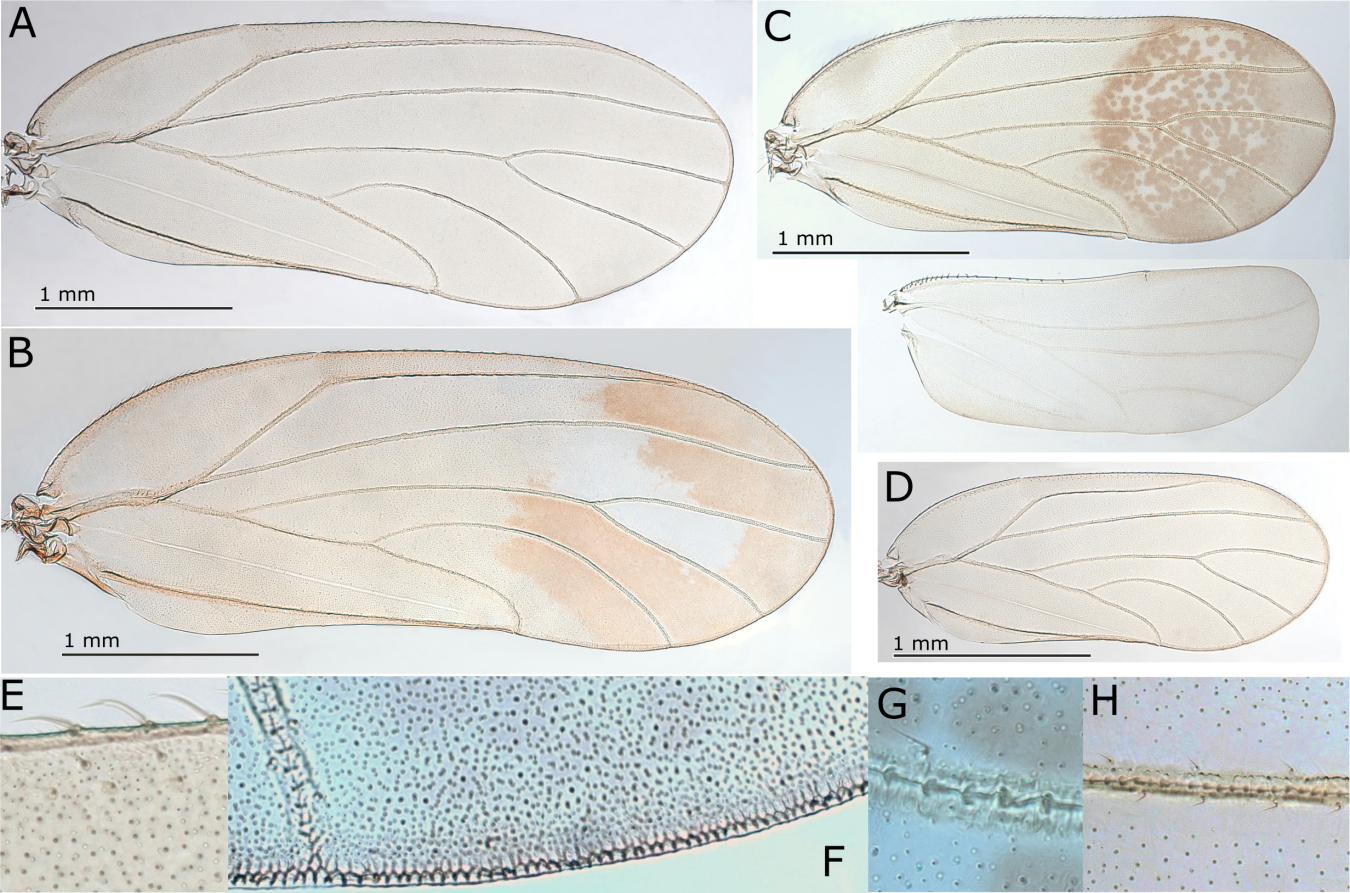
*Australopsylla* spp., forewings. **A**. *A. carinata*, female; **B**. *A. revoluta*, female; **C–H**. *A. exotica* Matsunaga & Percy, sp. nov.: **C**. Female forewing with hindwing below; **D**. Male; **E**. Setae on costal margin; **F**. Marginal spinule density (no radular spines); **G**. Vein detail showing transverse ridges; **H**. Spinule-free bands along veins in anterior portion.

### Annotated checklist of adventive psyllids in the Hawaiian Islands

#### Family Aphalaridae Löw, 1879

**Subfamily Aphalarinae Löw, 1879**

##### Aphalara rumicis

Taxon classificationAnimaliaHemipteraAphalaridae

Mally, 1894

069185A3-7B9E-5270-B4AD-CCC4B247132D

###### First detection/collection in HI.

1947 (Tuthill 1948).

###### Distribution in HI.

?O‘ahu (see Remarks).

###### Material examined.

No material located.

###### Host plant in HI.

Unknown.

###### Additional distribution and host plant records.

Continental USA, Canada; *Rumex altissimus* (Polygonaceae) (Burckhardt and Lauterer 1997).

###### Remarks.

Tuthill (1948) reported taking a single specimen “on a ridge overlooking Nuuanu Valley, Oahu on March 30, 1947”. Zimmerman (1957) referenced Tuthill’s find, listing *Aphalara rumicis* as the first introduced psyllid species in HI. Listings of *A. rumicis* for HI in Hodkinson (1983), Burckhardt and Lauterer (1997) and Nishida (2002) almost certainly cite the record from Tuthill. Tuthill’s single specimen could not be located. No other published records or specimens collected in HI could be found; hence, establishment is highly unlikely. Notably, there are several native *Rumex* species in HI, but no psyllids have been recorded from these.

###### Genetic resources.

None.

#### Subfamily Spondyliaspidinae Schwarz, 1898

##### Australopsylla

Taxon classificationAnimaliaHemipteraAphalaridae

Tuthill & Taylor, 1955

DD1B2FC1-9724-534F-AA8C-F4A2A48AB0AA

###### Comment and diagnosis.

*Australopsylla* is a genus native to Australia that has had little taxonomic treatment since the original descriptions by Froggatt (1900). Froggatt described two species, *Aphalara carinata* Froggatt, 1900 and *Rhinocola revoluta* Froggatt, 1900, which were subsequently transferred to a new genus, *Australopsylla*, by Tuthill and Taylor (1955). One species of *Australopsylla* is found in the Hawaiian Islands and is described here as a new species. All species are native to Australia and occur on *Eucalyptus* spp. (Hollis 2004; Martoni et al. 2024), but neither of the previously described species has been reported from *Eucalyptus robusta*, which is considered the primary host of *A. exotica* Matsunaga & Percy, sp. nov. (at least in the Hawaiian Islands). *Australopsylla* belongs to a clade that forms a well- supported group within Spondyliaspidinae (Percy et al. 2018; Fig. 15). The shape of the head in *Australopsylla*, with broadly truncate genal processes that are almost rectangular and a distinct preocular tubercule, is similar to *Blepharocosta* Taylor, *Cardiaspina* Crawford, *Creiis* Scott, and *Lasiopsylla* Froggatt (Tuthill and Taylor 1955; Taylor 1992a, 1992b). The shape of the male proctiger, including the much shorter apical portion, resembles *Blepharocosta* (Taylor 1992a); the paramere that is wide apically, often with a row of blunt peg-like teeth along the apical margin, is also present in some *Creiis*, *Dasypsylla* Froggatt, *Lasiopsylla* and *Cardiaspina* (Froggatt 1900; Taylor 1992b). All of these groups have immatures that produce lerps variously resembling bivalve-type shells. However, *Australopsylla* is unique among these groups in having sexually dimorphic forewings in some species, where pattern is present in females and absent in males (*Blepharocosta* species may have pattern difference in male and female forewings, but patterns are either present or absent in both sexes; Taylor 1992a).

###### Note.

Descriptions by Froggatt (1900) and Tuthill and Taylor (1955) lack many diagnostic characters required for reliable species identification, and no species key has previously been published. We therefore provide a key to the three described species of *Australopsylla* at the end of the checklist.

##### Australopsylla exotica

Taxon classificationAnimaliaHemipteraAphalaridae

Matsunaga & Percy sp. nov.

6F298CD6-12E8-5A6A-8ABD-44EDDDCC448A

https://zoobank.org/0DBD09F0-B3F4-4F65-A647-3FE253E60996

**Figure 8.**
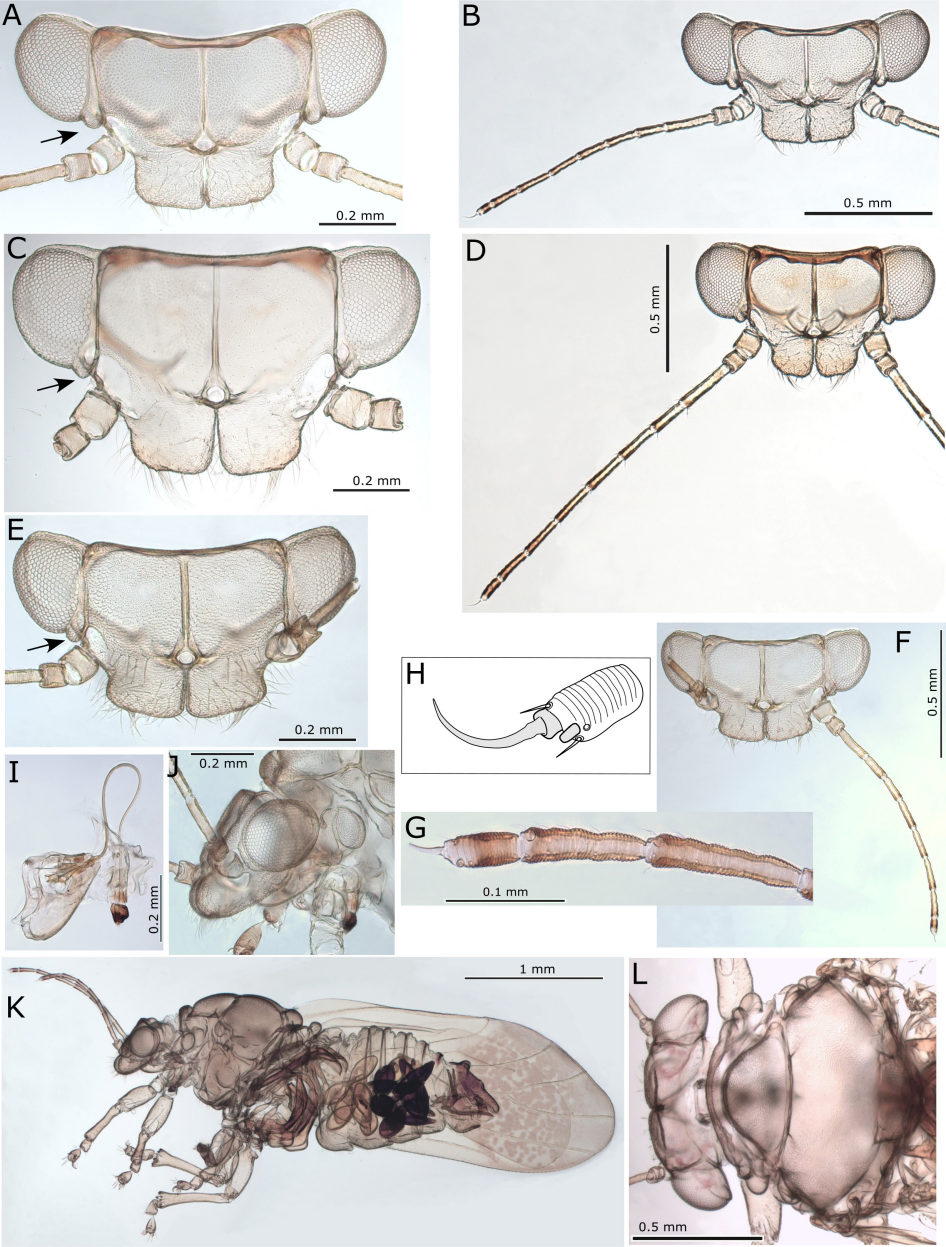
*Australopsylla* spp. **A, B**. *Australopsylla carinata*: **A**. Head (preocular tubercule arrowed); **B**. Head and antenna; **C, D**. *A. revoluta*: **C**. Head (preocular tubercule arrowed); **D**. Head and antenna; **E–L**. *A. exotica* Matsunaga & Percy, sp. nov.: **E**. Head (preocular tubercule arrowed); **F**. Head and antenna; **G**. Terminal antennal segments; **H**. Antennal segment 10 (two unequal terminal setae in grey); **I**. Clypeus and proboscis; **J**. Head and proboscis (lateral view); **K**. Female in lateral view; **L**. Head and thorax in dorsal view.

**Figure 9.**
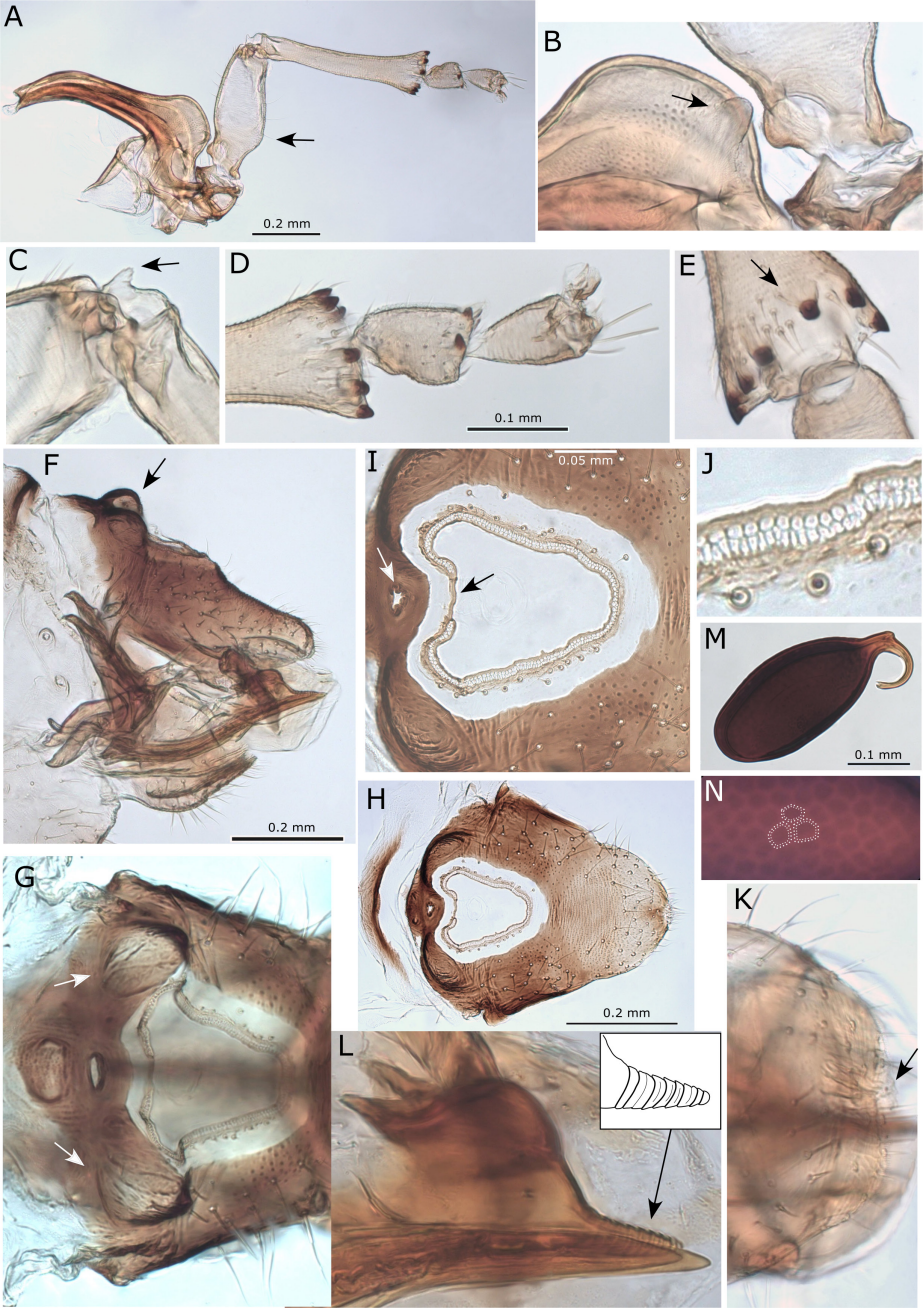
*Australopsylla exotica* Matsunaga & Percy, sp. nov.: **A**. Hind leg showing metafemur sub-basal inflation (arrow); **B**. Coxa swelling with blunt tubercle (arrow); **C**. Genual spine (arrow); **D**. Apex of metatibia and metatarsus; **E**. Apex of metatibia with isolated spur (arrow); **F**. Female terminalia in lateral view showing anterior proctiger humps (arrow); **G**. Female proctiger in dorsal view showing placement of humps (arrows) relative to anal ring; **H**. Female proctiger in dorsal view; **I**. Anal ring in dorsal view showing anterior pore (white arrow) and anal ring mid-anterior discontinuity (black arrow); **J**. Detail of anal ring pores; **K**. Female subgenital plate indicating cleft apex (arrow); **L**. Ovipositor with inset detail of ridges on dorsal valves; **M**. Egg; **N**. Honeycomb sculpturing on egg surface.

Figs 7C–H, 8E–L, 9A–K, 11A–E, 12A–K, 13A–F

###### Type locality.

USA, Hawaiian Islands, O‘ahu, Hale‘iwa; 21.6440°N, 158.0355°W.

###### Type material.

***Holotype***: USA – Hawaiian Islands • ♂; O‘ahu, Hale‘iwa; 21.6440°N, 158.0355°W; 29 Mar. 2019; A. Tateno, J. Yalemar & D. Cho leg.; ex *Eucalyptus robusta*; coll. 2019-056; slide-mounted; BPBM. ***Paratypes***: USA – Hawaiian Islands • 1 ♂, 3 immatures; as for holotype; slide-mounted; BPBM • 1 ♂, 4 ♀; O‘ahu; Wai‘anae Mountains, Ka‘ala Rd., 3^rd^ Gate; 21.5414°N, 158.1602°W; 01 Jun. 2021; K.N. Magnacca leg; ex *Eucalyptus* sp.; coll. 2021-125; slide-mounted; BPBM • 1 ♀; O‘ahu; southern Wai‘anae Mountains, Pālehua; 21.3954°N, 158.1024°W; 770 m; 23 Sep. 2021; K.N. Magnacca leg.; ex *Eucalyptus robusta*; coll. 2021-202b; slide-mounted; BPBM; • 2 ♀; O‘ahu; Palikea Trail; 21.404557°N, 158.098087°W; 23 Sep. 2021; J.N. Matsunaga & K.N. Magnacca leg; ex *Eucalyptus robusta*; coll. 2021-202a; slide mounted; BPBM.

###### Other material examined.

USA – Hawaiian Islands • 1 ♂, 1 ♀; O‘ahu, southern Wai‘anae Mountains, Pālehua; 21.3954°N, 158.1024°W; 770 m; 23 Sep. 2021; K.N. Magnacca leg.; ex *Eucalyptus robusta*; coll. 2021-202b; BPBM • 20 immatures, O‘ahu, Kāne‘ohe; 21.4133°N, 157.8004°W; 01 Jan. 2025; J.N. Matsunaga leg.; ex *Eucalyptus robusta*; BPBM • 2 ♂; O‘ahu, Wahiawā, Schofield Barracks Army Base; 21.4923°N, 158.0500°W; 12 Mar. 2019; A. Tateno, J. Yalemar & D. Niide leg.; ex *Eucalyptus robusta*; coll. 2019-043; BPBM • 2 ♀; Kaua‘i, Kōke‘e Road; 22.0380°N, 159.6665°W; 760 m; 28 Apr 2025; K.N. Magnacca leg.; sweeping *Eucalyptus* sp.; BPBM.

###### Diagnosis.

Morphologically, *A. exotica* can be distinguished from *A. carinata* by the presence of sexual dimorphism in forewing coloration, and from *A. revoluta*, which shares sexual forewing dimorphism with *A. exotica*, by the type and extent of pattern in the apical portion of female forewings (Fig. 7A–D). Additionally, *A. exotica* is a smaller species than the other two (Table 2). The structure of head, thorax, legs, and female terminalia are generally similar in all three species, with species-specific differences highlighted in the diagnostic key for *Australopsylla* adults (provided at the end of the checklist), as well as in Table 2 and the Systematics section for *A. exotica* (below).

###### Description.

***Adult color and structure***. General color light to mid-green, with some orange to orange-brown on the dorsum of thorax and head, and darker brown on the apical portion of the abdomen and terminalia of the female; forewing veins light brown, membrane ochreous, uniformly so in males, but with a distinctly darker brown apical third of forewing in females consisting of brown spots and a brown band around the apical margin (Fig. 13A, B). Head narrower than, or equal to, width of thorax, deflexed downwards ca 50–60° from longitudinal axis of body (Figs 8K, 13A); coronal suture distinct; occipital sclerite developed into a preocular tubercule (Fig. 8E); genal processes well developed and contiguous for most of the length, shorter than vertex length, broad and bluntly rounded in lateral view, in dorsal view somewhat rectangular and angular laterally (Fig. 8E, J). Distal proboscis segment extremely short (Fig. 8I). Antenna 10-segmented, longer than head width (AL:HW 1.2–1.4) (Fig. 8F), segment 3 longest, length of segments 4–9 subequal, segment 10 ~ 0.5× length of segment 9, a single subapical rhinarium on each of segments 4, 6, 8, and 9, and one or two small rhinaria apically on segment 10 (and possibly segment 5); segment 10 with a single terminal seta as long as segment, and a short blunt seta subapically (Fig. 8G, H). Thorax well arched (Figs 8K, 13A). Legs relatively short and stout (Fig. 9A); hind leg coxa swollen into a rectangular projection with small blunt tubercle, but without distinct meracanthus (Fig. 9A, B); metafemur short, slightly inflated sub-basally (Fig. 9A); metatibia length subequal to head width, longer than metafemur, with prominent basal genual spine (Fig. 9C), inflated apically with a crown of 6(7) sclerotized spurs arranged in two groups (Fig. 9D), one spur often more petiolate and separated from other spurs by unsclerotized setae (Fig. 9E); metatarsal segments subequal in length, proximal segment with two lateral spurs (Fig. 9D). Forewing length 2.7–3.7× head width, more or less parallel-sided, slightly wider apically, apex broadly rounded (Fig. 7C, D); costal break present, pterostigma well developed (length ~ 0.4× forewing length); costal margin with fringe of short setae (Fig. 7E) and minute setae bordering veins (Fig. 7H), veins with transverse ridges (Fig. 7G); vein Rs length ~ 0.7× wing length, slightly curved medially towards fore margin, vein M longer than its branches, veins M+Cu and Cu subequal in length, both cells cu_1_ and m_1_ elongate and narrow, vein Cu_1a_ strongly arched, vein Cu_1b_ termination curved to wing margin (Fig. 7C, D); membrane with surface spinules present in all cells, densely distributed throughout cells at apex (Fig. 7F), more sparsely distributed anteriorly with spinule-free bands along veins (Fig. 7H), lacking radular spine clusters. Hindwing broad, length ~ 0.8× forewing length, costal margin slightly sinuate, venation prominent, apex broadly rounded (Fig. 7C below). Male terminalia as in Fig. 11A–E; proctiger two-segmented, total length 1.5× length of paramere, basal segment, in lateral view more or less parallel-sided, hardly produced posteriorly, apical segment short, tubular, length ~ 0.3× length of basal segment; subgenital plate rounded, in lateral view with dorsal margin sinuous (Fig. 11A); paramere, in lateral view, wedge-shaped, narrowest at the base, expanding evenly and gradually to apex, exterior surface convex, interior surface concave with a row of sclerotized pegs along the apical margin, exterior surface with short simple setae, interior surface with longer, sometimes stout, simple setae, in posterior view, curving outwards in basal portion and inwards towards apex (Fig. 11B–D). Aedeagus distal segment short, pear-shaped, expanding towards apex, apex rounded bearing a recurved spine dorsally (Fig. 11E). Female terminalia as in Fig. 9F, short; proctiger 0.6× head width, dorsal surface with two raised humps anterior to and either side of anal ring, and a small, irregularly rounded pore in depression between humps anterior of anal ring, apex bluntly rounded (Fig. 9F–I); anal ring relatively large (length 0.3× proctiger length), heart-shaped, composed of a double row of cells that are discontinuous for a short, mid-anterior section (Fig. 9I); subgenital plate very short (length 0.5× proctiger length), apex blunt and slightly cleft medially (Fig. 9K); ovipositor dorsal valves apices with a series of ridges (Fig. 9L).

***Immature color and structure***. Fifth instar yellow-green, younger instars yellow-orange, all instars with dark brown to black sclerites, wing pads, legs, and terminal antennal segments (Fig. 13C, D). Fifth instar: body ~1.2–1.5× as long as wide, abdomen wider than head; wing pads protruding, lacking distinct humeral lobes; scattered, simple, short to medium-length setae throughout; thoracic and abdominal sclerites reduced but with larger, irregular shaped sclerites anterior to anus (Fig. 12I–K). Antenna length subequal to head width, 10-segmented with a single subapical rhinarium on each of segments 4, 6, 8, and 9, and two smaller rhinarium on segment 10 towards the apex, segment 3 longest, 6–10 subequal with 4 and 5 shorter, apex of segment 10 acute and bearing two short, unequal length setae (Fig. 12C). Several notable appendages found on wing pads and sclerites of thorax and abdomen as follows: two digitate appendages on wing pads, one on exterior of forewing pad and one interior at junction of forewing and hindwing pads; thorax with a pair of tubercles on sternites at base of middle legs and two well developed appendages ventrally on sternites near base of hind legs; abdomen with a pair of tubercles dorsally on tergites of first abdominal segment (Fig. 12E). Tarsi with no visible arolium, possibly a small knob-like unguitractor, claws small but well developed (Fig. 12H). Abdomen with 3+3 lateral pore fields ventrally on segments 3 and 4, consisting of two uneven lines of rounded pores, anterior pores mostly on tubular stalks and posterior pores on surface (Fig. 12A, K), third pore field smaller and adjacent to anal sclerite (Fig. 12K). Anus terminal, acute, surrounded by stout setae with fringed apices ventrally (Fig. 12K) and a fringed circular orifice dorsally (Fig. 12I, J).

The lateral pore fields and most appendages described for 5^th^ instar are clearly visible in 1^st^ instar onwards (Fig. 12G); exceptions are those on thoracic sternites, which are evident by 2^nd^ instar (though minute), and the exterior forewing pad appendage, which is evident by 3^rd^ instar. Antennae of 1^st^ instar 7-segmented, 2^nd^–3^rd^ instars 9-segmented and 4^th^ instar 10-segmented.

***Egg color and structure***. Narrowly ovoid, dark brown with honeycomb surface sculpturing, short basal pedicel, apparently lacking apical filament (Fig. 9M, N).

###### Measurements (mm) and ratios.

***Adults*** (3 males, 6 females) WL 2.25–3.03; WW 0.90–1.20; HW 0.74–0.88; AL 0.98–1.16; PB 0.06–0.07; WL:WW 2.43–2.63; WL:RsL 1.30–1.43; CUR 1.14–1.43; MR 0.42–0.52; HW:VW 1.66–1.75; VW:VL 1.81–2.05; VL:GC 1.62–2.08; WL:HW 2.71–3.65; AL:HW 1.26–1.40; HW:HT 1.48–1.68; HT:HF 1.47–1.92. Male terminalia: MP 0.25–0.27; PL 0.18; AEL 0.20; PL:HW 0.24; MP:PL 1.50; PL:SH 0.90. Female terminalia: FP 0.48–0.56; FSP 0.23–0.29; RL 0.14–0.17; OVH 0.06;; FP:RL 3.25–3.43; FP:HW 0.58–0.64; FP:SP 1.93–2.22.

***Immatures*** (5^th^ instar, *n* = 3): BL 1.84–2.27; BW 1.49–1.50; WPL 0.70–0.74; RW (orifice) 0.05–0.06; HW 0.82–0.90; AL 0.77–0.81; BL:BW 1.23–1.52; AL:HW 0.90–0.98.

***Eggs*** (dissected from abdomen, 4 females): EL 0.23–0.28; EW 0.13–0.15; EL:EW 1.77–1.93.

###### Etymology.

Named for the exotic status in the Hawaiian Islands where the species was recorded first. Adjective in the nominative singular.

###### Distribution.

Hawaiian Islands, Kaua‘i and O‘ahu; specimens first collected on O‘ahu in 2018, and the first record on Kaua‘i in 2025. *Australopsylla exotica* sp. nov. is Australian in origin, with known distribution in Queensland (see Systematics below). *Australopsylla exotica* was first reported infesting *Eucalyptus robusta* trees on a military base in Wahiawā by arborists and has since been found throughout *E. robusta* plantings in the Wai‘anae Mountain Range (JNM observations) as well as discovered in a residential area of Kāne‘ohe on a single *E. robusta* tree in 2023.

###### Host plant and biology.

Adults and immatures were collected from *Eucalyptus robusta*; some adults were also collected from *E. camaldulensis* and unidentified *Eucalyptus* spp. but *E. robusta* appears to be the preferred host. Eggs are laid at the base of leaf bud petioles (Fig. 13E). Emerging immatures settle on young shoots and produce lerps on the petioles and developing leaf buds (Fig. 13E), with aggregations often in quantities that cause shoot senescence (Fig. 13F). The lerps are brown, more or less smooth surfaced, rounded and shaped like a bivalve shell; as they enlarge they become clustered and overlapping (Fig. 13E, F).

The two *Australopsylla* species described to date from Australia are recorded producing lerps and leaf roll on the leaf blade of young and mature leaves of *Eucalyptus* spp. (Froggatt 1900). The lerps of *A. exotica* appear similar to the shell-like lerps described for these species and particularly similar in shape to those of *A. revoluta* (figured in Froggatt 1900), although the darker brown coloration appears more similar to that described for *A. carinata*, while *A. revoluta* lerps are described as white (Froggatt 1900).

###### Systematics.

**Figure 10.**
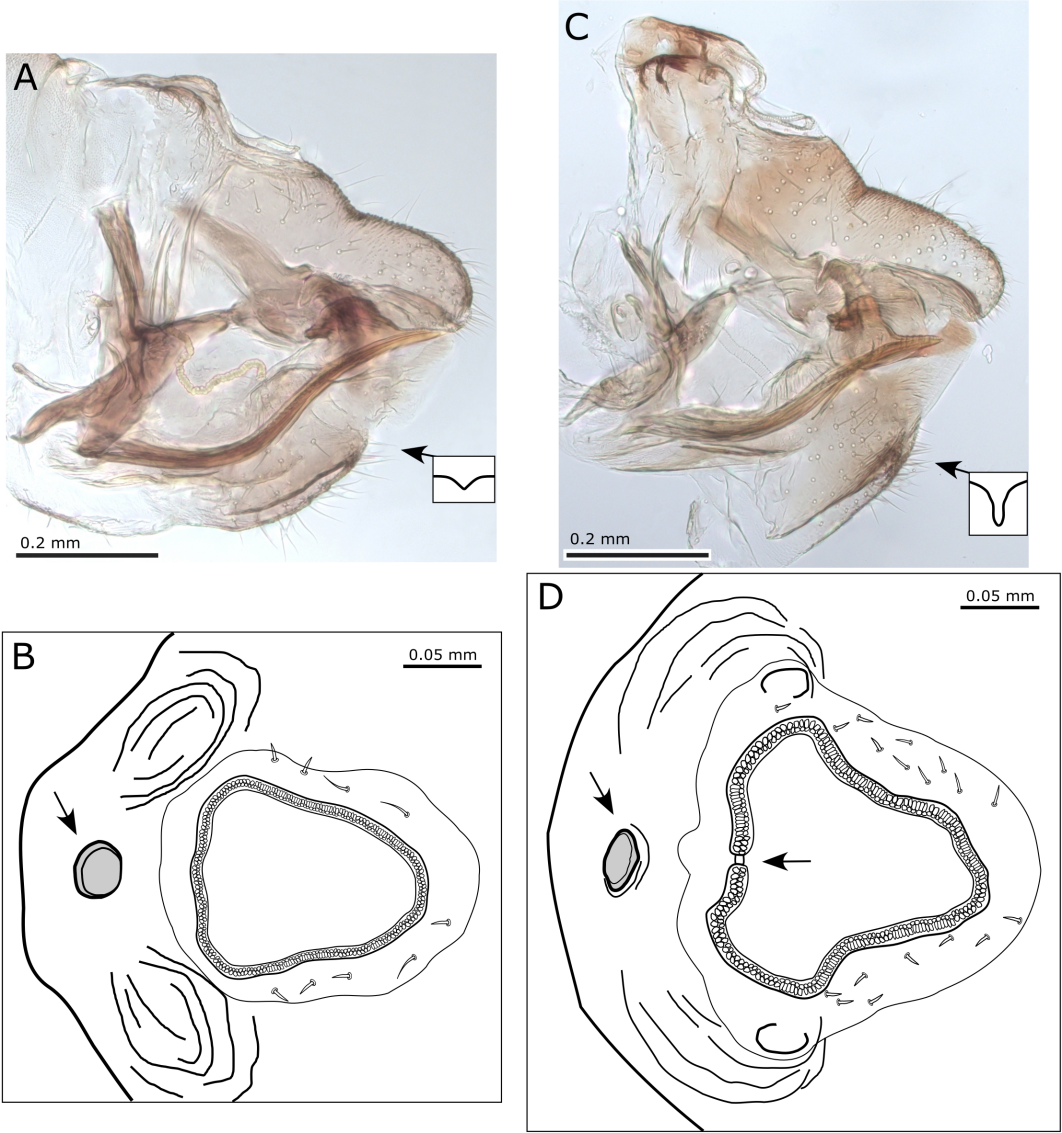
*Australopsylla* spp. **A, B**. *A. carinata*: **A**. Female terminalia in lateral view, inset medial cleft on apex of subgenital plate; **B**. Anterior portion of female proctiger in dorsal view showing anterior pore (arrow) and anal ring shape with position relative to anterior humps; **C, D**. *A. revoluta*: **C**. Female terminalia in lateral view, inset medial cleft on apex of subgenital plate; **D**. Anterior portion of female proctiger in dorsal view showing anterior pore (arrow) and anal ring shape with position relative to anterior humps, and short mid-anterior discontinuity in anal ring (arrow).

The mitogenomic data presented by Percy et al. (2018) places *Australopsylla* as sister to *Creiis*/*Lasiopsylla*, and these in turn as sister to *Cardiaspina*, which confirms the assessment by Tuthill and Taylor (1955) that *Australopsylla* “lies between *Creiis* and *Cardiaspina*” (Tuthill and Taylor 1955). Although not included in the study of Percy et al. (2018), the genus *Blepharocosta* is another endemic Australian *Eucalyptus*-feeding genus (Martoni et al. 2024) that is likely close to *Australopsylla* (Taylor 1992a). Since discovery of *Australopsylla exotica* sp. nov. in the Hawaiian Islands in 2018, a light trapping survey in Queensland, Australia listed “*Blepharocosta* sp. A” but without any morphological details (Martoni et al. 2020). Subsequent sequences for this taxon (F. Martoni pers. comm.) on GenBank (MT375255-57) were corrected by the original authors to “*Australopsylla* sp. A”. We have compared DNA data for “*Australopsylla* sp. A” with *A. exotica* and confirm these samples are conspecific, which therefore confirms the origin for *A. exotica* as Australia with native distribution in Queensland. Maximum genetic divergence using COI sequences of these GenBank samples from Queensland and the Hawaiian Islands is 0.6%. Analysis of just COI data, as well as placement of *A. exotica* (using COI and cytB data) in the mitogenome tree using the RAxML backbone constraint analysis, shows that *A. exotica* is more closely related to *A. revoluta*, with *A. carinata* sister to these taxa (Fig. 15). Maximum genetic distance between *A. exotica* and *A. revoluta* (the type species of *Australopsylla*) is 15.3%, and these in turn to *A. carinata* show 16.1–18.9% genetic divergence. The shared sexual forewing dimorphism in *A. exotica* and *A. revoluta* also supports a closer relationship between these two species than either to *A. carinata*; however the overall forewing shape is shorter and broader in *A. exotica*, moreover *A. carinata* and *A. revoluta* share an Rs vein more curved medially towards the fore margin (only slightly so in *A. exotica*), longer and narrower cell m_1_ (shorter and wider in *A. exotica*) and gradually tapering pterostigma (abruptly narrowing at apex in *A. exotica*) (Fig. 7). The shape of the head is similar in all three species (all have a distinct preocular tubercule), but the antennae are comparatively longer in *A. revoluta* (Fig. 8A–F, Table 2). The female terminalia are similarly short in all three species, which share a proctiger with distinct anterior humps and rounded pore anterior to anal ring, but in *A. carinata* the anal ring does not have a discontinuous mid-anterior section (which is short in *A. revoluta*, longer in *A. exotica*), and does not extend outwards and upwards on the humps as in *A. exotica* and *A. revoluta*; in addition, the female subgenital plate has a deeper cleft apically in *A. revoluta* (Fig. 10A, C); all three species have similarly serrated dorsal ovipositor valves (Figs 9, 10).

**Figure 11.**
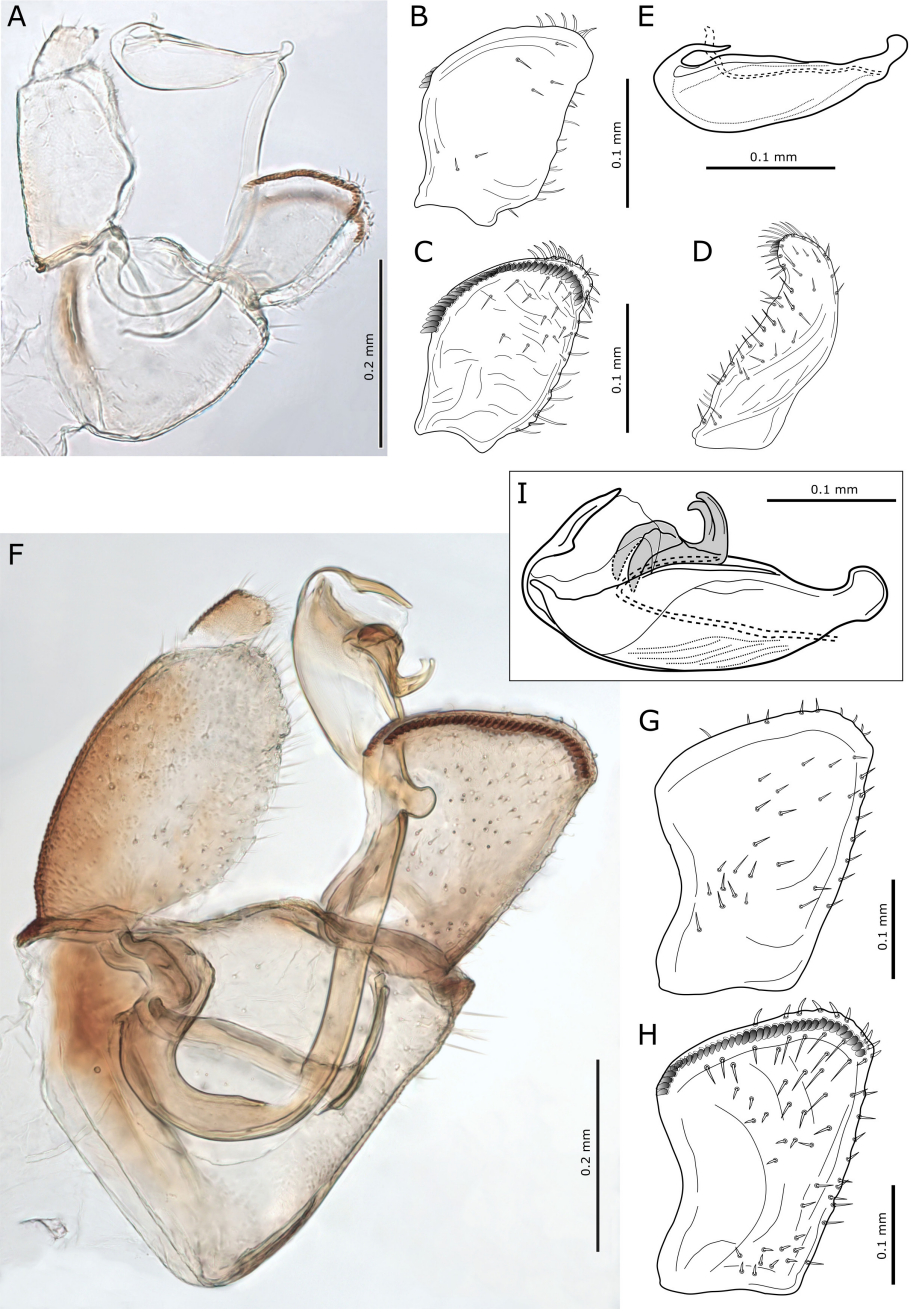
*Australopsylla* spp. **A–E**. *A. exotica* Matsunaga & Percy, sp. nov.: **A**. Male terminalia in lateral view; **B**. Paramere in external view; **C**. Paramere in interior view; **D**. Paramere in posterior view; **E**. Aedeagus distal segment; **F–I**. *A. revoluta*: **F**. Male terminalia in lateral view; **G**. Paramere in interior view; **H**. Paramere in posterior view; **I**. Aedeagus distal segment with mid-dorsal projection (in grey).

**Figure 12.**
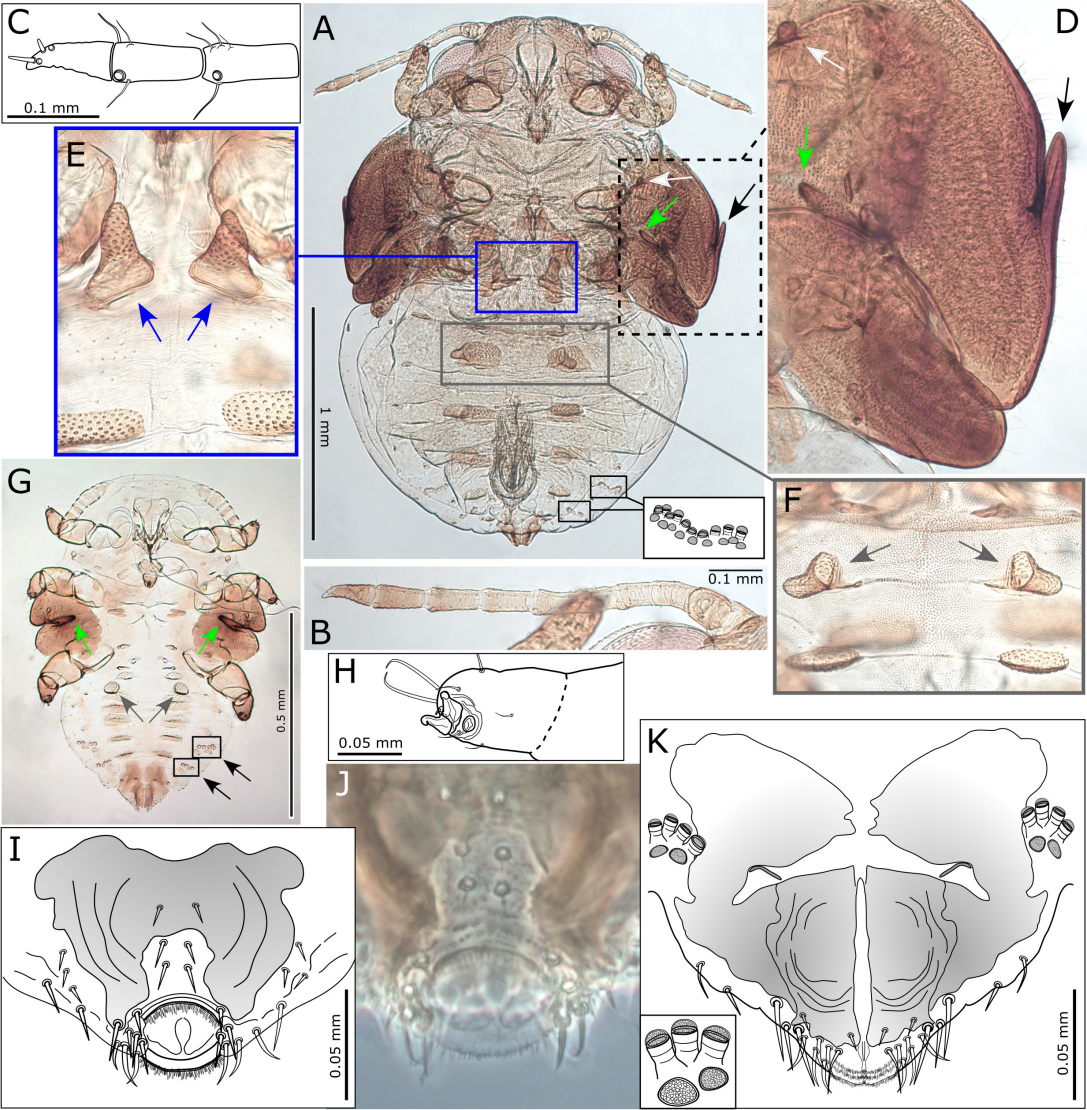
*Australopsylla exotica* Matsunaga & Percy, sp. nov., immatures: **A**. 5^th^ instar immature indicating position of appendages – on sternites (white arrow, blue box), on tergites (brown box), on wing pads (green and black arrows), and lateral position of abdominal pore fields; **B**. 5^th^ instar antenna; **C**. Detail of terminal antennal segments; **D**. Detail of interior (green arrow) and exterior (black arrow) digitate wing pad appendages and sternite tubercle at base of middle legs (white arrow); **E**. Detail of sternite tubercles at base of hind legs; **F**. Detail of tergite tubercles on 1^st^ abdominal segment; **G**. 1^st^ instar showing relative positions of wing pad interior digitate appendages (green arrows), tergite tubercles (brown arrows) and lateral abdominal pore fields; **H**. Tarsal claws; **I, J**. Dorso-posterior view of anus showing oval orifice; **K**. Ventro-posterior view of anus with inset detail of stalked and unstalked pores.

**Figure 13.**
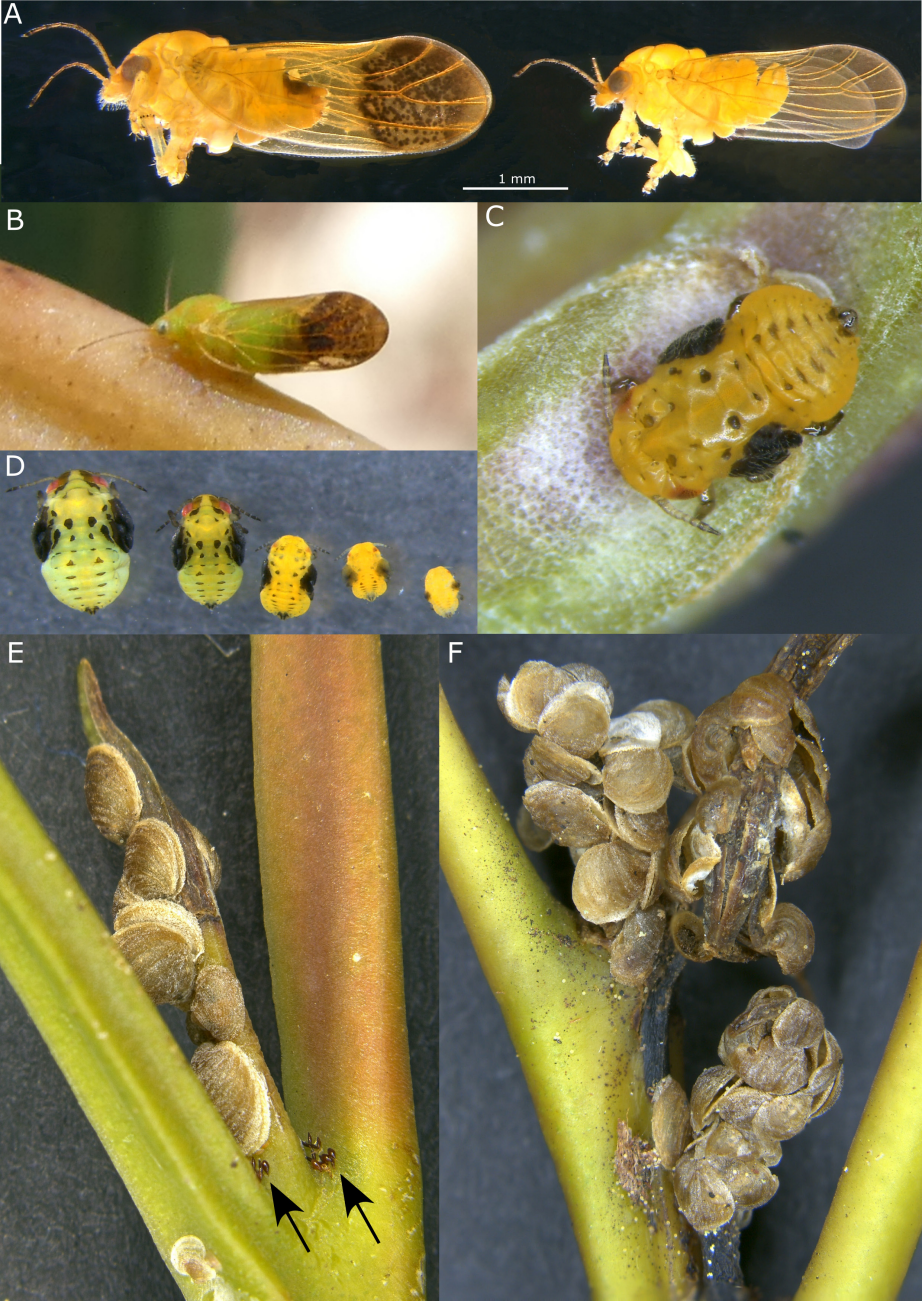
*Australopsylla exotica* Matsunaga & Percy, sp. nov. **A**. Adult female (left) and male (right); **B**. Adult female on *Eucalyptus* stem; **C**. 3^rd^ instar on *Eucalyptus* stem; **D**. Instars 1–5 showing size and color variation; **E**. Eggs in axil of leaf bud petiole (arrows) and lerps on leaf bud; **F**. Crowded lerps on senescing leaf buds.

###### Genetic resources.

GenBank PX507502–PX507503 (COI), PX527527–PX527528 (cytB) [Hawaiian specimens] (Table 3); MT375255–MT375257 [Australian specimens].

##### Blastopsylla occidentalis

Taxon classificationAnimaliaHemipteraAphalaridae

Taylor, 1985

C3B8D28C-24F6-5FD3-BA14-C5F191341029

###### First detection/collection in HI.

1993 (Beardsley and Uchida 2001).

###### Distribution in HI.

Kaua‘i, O‘ahu, Lāna‘i (new island record), Maui (probable new island record – see Remarks).

###### Material examined.

USA – Hawaiian Islands **·** 1 ♂, 1 ♀; Kaua‘i, Upper Wailua River, State Forestry Arboretum; 3 Jun. 1993; J. Beardsley & G. Uchida leg.; ex *Eucalyptus grandis*; coll. 1993-234; HDAB**·** 8 adults; O‘ahu, Honolulu, UH Mānoa campus; 06 Jun. 1993; J.W. Beardsley leg.; ex *Corymbia citriodora*; UHIM**·** 2 adults, 2 immatures; Lāna‘i, Lāna‘i City, Koele Lodge; 01 May 1995; C. McGrath & G.K. Uchida leg.; ex *Eucalyptus* sp.; HDAB**·** 1 adult; O‘ahu, southern Wai‘anae Mtns, Palikea; 21.4134°N, 158.0999°W; 859 m; 08 Apr. 2010; spec. PKSP32083; P.D. Krushelnycky leg.; sweeping; PDK collection **·** 1 adult; O‘ahu, northern Wai‘anae Mtns., Kahanahāiki Valley; 21.54119°N, 158.19409°W; 580 m; 10 May 2012; spec. PKSP26258; P.D. Krushelnycky leg.; sweeping; PDK collection.

###### Host plants in HI.

*Eucalyptus camaldulensis*, *E. deglupta*, *E. grandis*, *E. robusta*, *Eucalyptus* sp., *Corymbia citriodora* (Myrtaceae).

###### Additional distribution and host plant records.

Native to Australia and adventive worldwide, including continental USA; *Eucalyptus* spp. (Myrtaceae) (Halbert and Burckhardt 2020; Duarte et al. 2025; Martoni et al. 2025).

###### Remarks.

Previously recorded from Kaua‘i and O‘ahu (Beardsley and Uchida 2001). The Maui record (1994) appears in HDOA unpublished data; however, specimens could not be tracked. Immatures are free-living.

###### Genetic resources.

GenBank e.g., MG988657 (COI), MG988950 (cytB) [Norfolk Island specimen]. A number of other accession numbers for this species can be found on GenBank.

##### Cryptoneossa triangula

Taxon classificationAnimaliaHemipteraAphalaridae

Taylor, 1990

2325DDB4-5C95-5A41-9E45-157091B75DF6

###### First detection/collection in HI.

2018 (new state record; see Remarks).

###### Distribution in HI.

O‘ahu, Hawai‘i.

###### Material examined.

USA – Hawaiian Islands • 2 ♂, 1 immature; Hawai‘i, Keāhole, Kona International Airport; 19.7311°N, 156.0410°W; 22 May 2018; S. Chun & M. Fukada leg.; ex *Corymbia citriodora*; coll. 2018-129; USNM • 2 ♂, 1 ♀, 2 immatures; same data as previous; HDAB • 2 ♀, immatures; Hawai‘i, Keāhole, Kona International Airport; 19.7311°N, 156.0410°W; 30 May 2018; C. Okamoto leg.; ex *Corymbia citriodora*; coll. 2018-195 USNM • 1 ♂, 1 ♀, 1 immature; same info as previous; HDAB • 3 ♂, 2 ♀; O‘ahu, ‘Aiea Loop Trail; 16 Feb 2025, K.N. Magnacca leg.; sweeping *Corymbia citriodora*, BPBM.

###### Host plant in HI.

*Corymbia citriodora* (Myrtaceae).

###### Additional distribution and host plant records.

Native to Australia, adventive in other Pacific regions, including western USA; *Corymbia* spp. (Myrtaceae) (Percy et al. 2012; Castillo Carrillo et al. 2016; Martoni et al. 2025).

###### Remarks.

First discovered on lemon-scented gum trees planted around the Kona International Airport (island of Hawai‘i) while examining lerps of another species. Immatures are free-living, but often colonize the lerps of other species and were found under empty *Eucalyptolyma maideni* lerps. Identification confirmed by Cheryle O’Donnell (USNM). First discovered on O‘ahu in 2025.

###### Genetic resources.

GenBank e.g., MG195336 (COI) [Australian specimen]. A number of other accession numbers for this species can be found on GenBank.

##### Ctenarytaina eucalypti

Taxon classificationAnimaliaHemipteraAphalaridae

(Maskell, 1890)

0E0008B0-F92F-50CC-9674-198737183469

###### First detection/collection in HI.

1993 (Kumashiro et al. 2002).

###### Distribution in HI.

Maui, Hawai‘i.

###### Material examined.

USA – Hawaiian Islands • ~ 20 adults; Maui, East Maui, Makawao, start of Olinda Road; 20.8037°N, 156.2766°W; 830 m; 03 Jul. 2014; D.M. Percy leg.; ex *Eucalyptus* sp.; coll. Hi500-14; DMPC • 1 ♂, 1 ♀; Hawai‘i, Kamuela, DLNR tree nursery; 12 Jul. 1993; J. Lum leg.; ex *Eucalyptus* sp.; coll. 1993-277; HDAB • 1 ♂, 1 ♀; Hawai‘i, Kamuela; 04 Aug. 1994; S. Matayoshi leg.; ex *Eucalyptus globulus*; coll. 1994-545; HDAB.

###### Host plant in HI.

*Eucalyptus globulus* (Myrtaceae).

###### Additional distribution and host plant records.

Native to Australia, adventive in western USA, and many other locations worldwide; *Eucalyptus* spp. (Myrtaceae) (Burckhardt et al. 1999; Castillo Carrillo et al. 2016; Halbert and Burckhardt 2020; Duarte et al. 2025).

###### Remarks.

Collected in several locations in East Maui and Kamuela, Hawai‘i. Immatures are free-living.

###### Genetic resources.

GenBank PX507506–PX507507 (COI), PX527531–PX527532 (cytB) (Table 3), SRX1277825 (transcriptome) [Hawaiian specimens]. A number of other accession numbers for this species can be found on GenBank.

##### Ctenarytaina longicauda

Taxon classificationAnimaliaHemipteraAphalaridae

Taylor, 1987

AA1D9605-4BC8-562D-A15A-8BECDE0086FB

###### First detection/collection in HI.

2010 (new state record; see Remarks).

###### Distribution in HI.

O‘ahu.

###### Material examined.

USA – Hawaiian Islands **·** 1 ♂; O‘ahu, southern Wai‘anae Mountains, Palikea; 21.4107°N, 158.0983°W; 822 m; 08 Apr. 2010; spec. PKSP12326; P.D. Krushelnycky leg.; sweeping; DMPC**·** 1 ♀; O‘ahu, northern Ko‘olau Mountains, Pūpūkea; 21.6375°N, 158.0120°W; 315 m; 08 Jul. 2014; D.M. Percy leg; ex *Metrosideros*; coll. Hi76-14; DMPC**·** 3 ♂, 3 ♀; O‘ahu, southern Wai‘anae Mountains, Palikea; 21.403474°N, 158.097985°W; 23 Sept. 2021; J. Matsunaga & K. Magnacca leg.; ex *Lophostemon confertus*; coll. 2021-200; HDAB, DMPC**·** 2 adults; O‘ahu, Wai‘anae Mountains, Honouliuli Forest Reserve; 21.4635°N, 158.0931°W; 04 Oct. 2021; K. Magnacca leg.; ex *Lophostemon confertus*; coll. 2021-220; USNM.

###### Host plant in HI.

*Lophostemon confertus* (Myrtaceae).

###### Additional distribution and host plant records.

Native to Australia, adventive in New Zealand and California; *Lophostemon confertus* and possibly *L. suaveolens* (Myrtaceae) (Taylor 1987b; Hollis 2004; Percy et al. 2012; Martoni et al. 2016).

###### Remarks.

Collected in the northern Ko‘olau Mountains and southern Wai‘anae Mountains, O‘ahu. PDK identified specimens collected from the Palikea area in 2010, and identifications were confirmed by Cheryle O’Donnell (USNM). Immatures are free-living.

###### Genetic resources.

GenBank PP218312–PP218313, PX507508 (COI), PP235413–PP235414, PX527533 (cytB) [Hawaiian specimens] (Table 3). A number of other accession numbers for this species can be found on GenBank.

##### Ctenarytaina spatulata

Taxon classificationAnimaliaHemipteraAphalaridae

Taylor, 1997

3A8CEAEB-3A03-5C76-890D-DB17242A4805

###### First detection/collection in HI.

2010 (new state record; see Remarks).

###### Distribution in HI.

Kaua‘i, O‘ahu, Hawai‘i.

###### Material examined.

USA – Hawaiian Islands **·** 1 ♀; O‘ahu, southern Wai‘anae Mountains, TNC Honouliuli Preserve, Palikea Trail; 21.4142°N, 158.0993°W; 900 m; 19–20 Jul. 2010; spec. PKSP31984; P.D. Krushelnycky leg.; sweeping; DMPC**·** 1 ♂, 2 ♀; O‘ahu, southern Ko‘olau Mountains, Mānoa Cliff Trail; 21.3452°N, 157.8048°W; 565 m; 10 Jul. 2014; D.M. Percy leg.; ex *Metrosideros*; coll. Hi84-14; DMPC**·** 1 ♂; O‘ahu, southern Wai‘anae Mountains, Palikea Trail; 21.4046°N, 158.0981°W; 23 Sep. 2021; J.N. Matsunaga & K. Magnacca leg.; ex *Eucalyptus robusta*; coll. 2021-201; HDAB**·** 3 adults; O‘ahu, southern Wai‘anae Mts., Mauna Kapū, Palikea Trail; 21.4046°N, 158.0980°W; 23 Sep. 2021; K. Magnacca leg.; ex *Eucalyptus robusta*; coll. 2021-221; USNM**·** 1 ♂, 2 ♀; Hawai‘i, Waiki‘i; 19.8590°N, 155.6500°W; 27 Jun. 2018; S. Chun leg.; ex *Eucalyptus camaldulensis*; coll. 2018-192; HDAB**·** 1 ♂, 3 ♀; Hawai‘i, Volcano; 13 May 2025; K. Magnacca leg.; sweeping *Eucalyptus camaldulensis*; BPBM**·** 1 ♀; Kaua‘i, Kōke‘e State Park; 22.114°N, 159.670°W; 23 Apr. 2021; K. Magnacca leg.; sweeping *Dodonaea viscosa*; coll. 2022-166; HDAB.

###### Host plant in HI.

Adults collected from *Eucalyptus robusta* and *E. camaldulensis* (Myrtaceae).

###### Additional distribution and host plant records.

Native to Australia, adventive in Europe, New Zealand, South America, USA; several different *Eucalyptus* spp., including *E. camaldulensis*, *E. globulus*, *E. grandis*, *E. parvifolia*, *E. viminalis* (Myrtaceae) (Percy et al. 2012; Halbert and Burckhardt 2020; Duarte et al. 2025).

###### Remarks.

Collected in southern Ko‘olau Mountains and southern Wai‘anae Mountains (O‘ahu), Waiki‘i (Hawai‘i), and Kōke‘e State Park (Kaua‘i). Identification confirmed by Cheryle O’Donnell (USNM). Immatures are free-living.

###### Genetic resources.

GenBank PX507509 (COI), PX527534 (cytB) [Hawaiian specimen] (Table 3). A number of other accession numbers for this species can be found on GenBank.

##### Eucalyptolyma maideni

Taxon classificationAnimaliaHemipteraAphalaridae

Froggatt, 1901

1455AA66-02C7-5CE4-9CC7-2F72CC09E877

###### First detection/collection in HI.

2018 (new state record; see Remarks).

###### Distribution in HI.

O‘ahu, ?Hawai‘i (see Remarks).

###### Material examined.

USA – Hawaiian Islands **·** Lerps only; Hawai‘i, Keāhole, Kona International Airport; 19.7311°N, 156.0410°W; 22 May 2018; S. Chun & M. Fukada leg.; ex *Corymbia citriodora*; coll. 2018-249; HDAB, USNM**·** Lerps only; Hawai‘i, Keāhole, Kona International Airport; 19.7311°N, 156.0410°W; 30 May 2018; C. Okamoto leg.; ex *Corymbia citriodora*; coll. 2018-250; HDAB**·** 2 ♂; O‘ahu, ‘Aiea Loop Trail; 16 Feb. 2025; K.N. Magnacca leg.; sweeping *Corymbia citriodora*; BPBM**·** 1 ♂, 2 ♀; O‘ahu, Makakilo Park; 24 May 2025; K.N. Magnacca leg.; sweeping *Corymbia citriodora*; BPBM.

###### Host plant in HI.

*Corymbia citriodora* (Myrtaceae).

###### Additional distribution and host plant records.

Native to Australia, adventive in California; *Corymbia* spp. (Myrtaceae) (Percy et al. 2012; Martoni et al. 2024).

###### Remarks.

**Figure 14.**
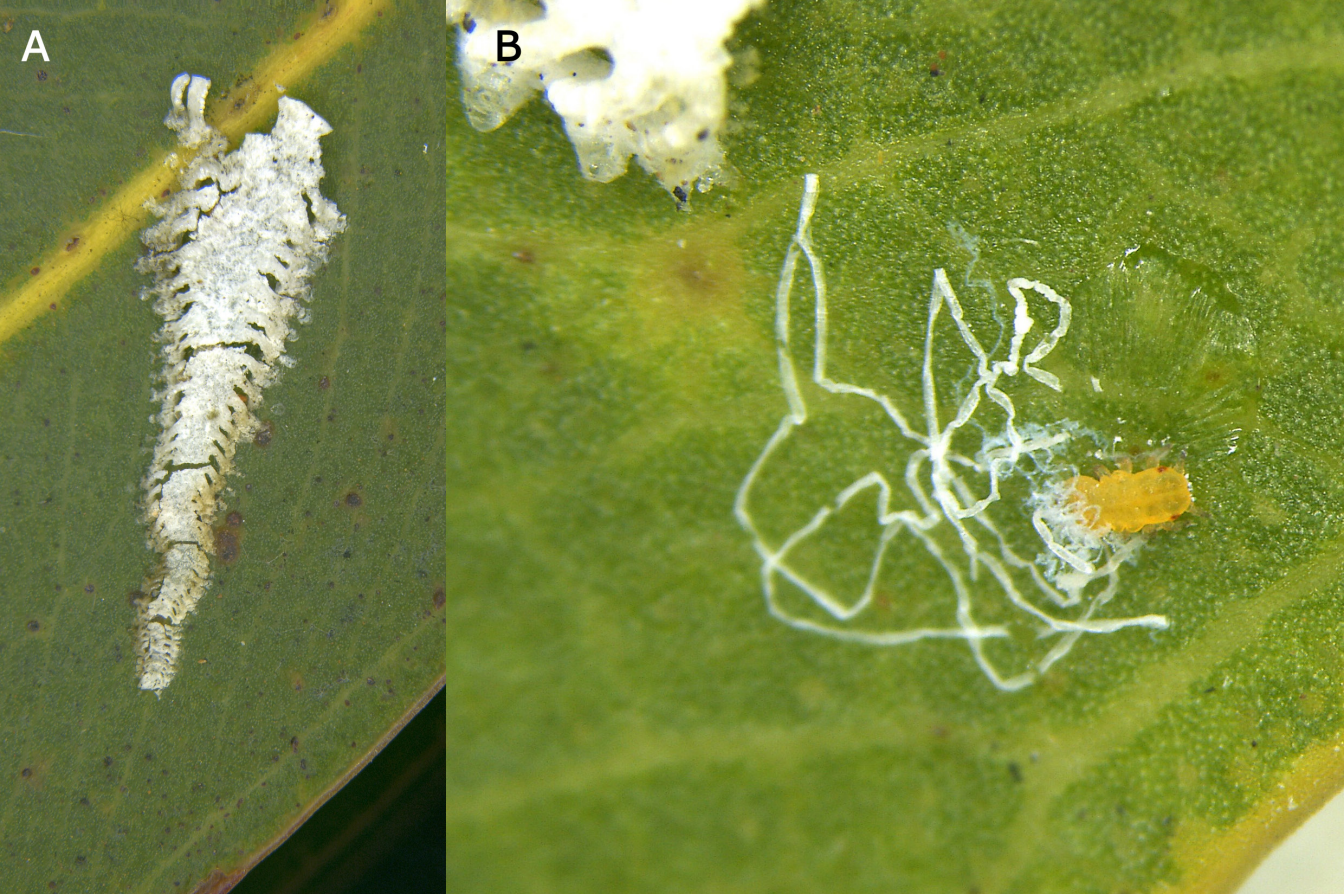
*Eucalyptolyma maideni*. **A**. Lerp on *Corymbia citriodora* leaf; **B**. Early instar immature producing waxy filamentous flocculence.

In 2018, characteristic waxy lerps on host tree leaves were observed on Hawai‘i (Fig. 14). However, only immature exuviae, predatory insects, and parasitized immatures were found. No adults or live immatures were collected. The lerps were comparable with those of *Eucalyptolyma maideni* (Taylor 1987a; Martoni et al. 2024). Then, in February and May 2025, adults were collected on O‘ahu. Additional sweeping of trees on which lerps were found on Hawai‘i did not locate specimens, and no lerps were visible upon re-inspection in August 2025. Immatures develop under lerps.

###### Genetic resources.

GenBank e.g., KY923934, MG132459 [Australian and New Zealand specimens]. A number of other accession numbers for this species can be found on GenBank.

##### Glycaspis brimblecombei

Taxon classificationAnimaliaHemipteraAphalaridae

Moore, 1964

14C12883-F309-565B-9B6F-E5CEAEAD8C50

###### First detection/collection in HI.

2001 (Nagamine and Heu 2001; Matsunaga et al. 2019).

###### Distribution in HI.

O‘ahu, Maui, Hawai‘i.

###### Material examined.

USA – Hawaiian Islands **·** 35 adults, immatures; Maui, Ulupalakua; 26 Apr. 2001; W. Nagamine leg.; ex *Eucalyptus camaldulensis*; coll. 2001-037; HDAB**·** 1 adult; O‘ahu, southern Wai‘anae Mountains, Palikea; 21.4143°N, 158.0994°W; 900 m; 18 Jul. 2012; spec. PKSP36031; P.D. Krushelnycky leg.; sweeping; PDK collection **·** 1 adult; Maui, Haleakalā NP; 20.7504°N, 156.2477°W; 2302 m; 10 Jul. 2003; spec. DS05040; P.D. Krushelnycky leg.; ex *Sophora chrysophylla*; PDK collection **·** 5 adults, nymphs; Hawai‘i, Waiki‘i; 19.8590°N, 155.6499°W; 27 Jun. 2018; S. Chun leg.; ex *Eucalyptus camaldulensis*; coll. 2018-191; HDAB.

###### Host plants in HI.

*Eucalyptus camaldulensis*, *E. robusta*, *E. cf. robusta* (Myrtaceae).

###### Additional distribution and host plant records.

Native to Australia, adventive in other regions of the world including Africa, North, Central, and South America, and Europe; *Eucalyptus* spp. (Myrtaceae) (Percy et al. 2012; Halbert and Burckhardt 2020; Martoni et al. 2024; Duarte et al. 2025).

###### Remarks.

Immatures develop under a whitish to greyish hemispherical lerp resembling an armored scale insect.

###### Genetic resources.

GenBank e.g., MW535997, OR068451 [European specimens]. A number of other accession numbers for this species can be found on GenBank.

#### Family Calophyidae Vondráček, 1957

**Subfamily Calophyinae Vondráček, 1957**

##### Calophya rubra

Taxon classificationAnimaliaHemipteraCalophyidae

(Blanchard, 1852)

F4CBD3AB-8334-5004-B292-8726AD5E0249

###### First detection/collection in HI.

1989 (Conant et al. 1992).

###### Distribution in HI.

Maui.

###### Material examined.

No material was located in HI collections.

###### Host plant in HI.

*Schinus molle* (Anacardiaceae).

###### Additional distribution and host plant records.

South America; *Schinus* spp. (Anacardiaceae), at least four different species (Burckhardt and Basset 2000; Ouvrard 2023).

###### Remarks.

Collected throughout the Kula area from Ulupalakua to Makawao and in Wailuku (Conant et al. 1992). Immatures are found in closed galls on twigs and branchlets (Burckhardt and Basset 2000).

###### Genetic resources.

None.

#### Family Carsidaridae Crawford, 1911

**Subfamily Carsidarinae Crawford, 1911**

##### Mesohomotoma hibisci

Taxon classificationAnimaliaHemipteraCarsidaridae

(Froggatt, 1901)

2DBBDA74-78B9-507F-9EBA-F60BEA8C6D9B

###### First detection/collection in HI.

2005 (Matsunaga et al. 2019).

###### Distribution in HI.

O‘ahu, Maui.

###### Material examined.

USA – Hawaiian Islands **·** 12 ♂, 8 ♀, 32 immatures; O‘ahu, Makiki; 20 May 2005; W. Nagamine leg.; ex *Hibiscus tiliaceus*; coll. 2005-044; HDAB, USNM**·** 9 ♂, 10 ♀, 30 immatures; Maui, Kahului, UH Maui College Campus; 20.8910°N, 156.4812°W; 3 Jan. 2020; M. Fukada leg.; ex *Hibiscus tiliaceus*; coll. 2020-025; HDAB.

###### Host plant in HI.

*Hibiscus tiliaceus* (Malvaceae).

###### Additional distribution and host plant records.

Native to Australia and Pacific Islands; known from Africa, Asia; *Hibiscus* spp. (Malvaceae) (Hollis 1987; Martoni et al. 2025).

###### Remarks.

Immatures are free-living but often produce abundant white flocculence.

###### Genetic resources.

GenBank e.g., MG989231 (complete mitochondrion), KY294172 [French Polynesia specimens]. A number of other accession numbers for this species can be found on GenBank.

#### Subfamily Homotominae Heslop-Harrison, 1958

##### Macrohomotoma gladiata

Taxon classificationAnimaliaHemipteraCarsidaridae

Kuwayama , 1908

A42BF994-A8DB-5B4B-AC87-4F54A334A289

###### First detection/collection in HI.

2022 (Matsunaga 2022).

###### Distribution in HI.

O‘ahu.

###### Material examined.

USA – Hawaiian Islands **·** 10 ♂, 10 ♀, 50 immatures; O‘ahu, Honolulu, Māpunapuna; 21.3376°N, 157.8975°W; 3 Nov. 2002; M. Ramadan leg.; ex *Ficus microcarpa*; coll. 2022-231; HDAB, USNM**·** 2 ♂, 30 immatures; O‘ahu, Honolulu, Kapālama; 21.3289°N, 157.8640°W; 4 Nov. 2022; D. Cho, J. Yalemar leg.; ex *Ficus microcarpa*; coll. 2022-234; HDAB**·** 1 ♂, O‘ahu, UH Mānoa campus; 11 May 2025; K.N. Magnacca leg.; on *Ficus benjamina*; BPBM**·** Immatures; O‘ahu, Kāne‘ohe; 21.4067°N, 157.8030°W; 5 Sep. 2025; J. Matsunaga leg.; ex *Ficus microcarpa*; JNM2025-01.

###### Host plant in HI.

*Ficus microcarpa* and possibly *F. benjamina* (only adults collected) (Moraceae).

###### Additional distribution and host plant records.

Native to China, India, Indonesia, Japan, Malaysia, and Taiwan; adventive in California and Mediterranean countries in southern Europe and North Africa; *Ficus* spp. (Moraceae) (Yang 1984; Mifsud and Porcelli 2012; Rung 2016; Ouvrard 2023).

###### Remarks.

First recorded for HI as *Pauropsylla apsylloides* by Crawford (1919), but only based on a single damaged specimen (possibly dead on arrival off a recently imported plant), and the taxon was not recorded thereafter as established. However, subsequent authors continued to cite the original record (e.g., Hodkinson 1983, 1986; Hollis and Broomfield 1989; Nishida 2002; Burckhardt et al. 2018). Subsequently placed in *Macrohomotoma* and later synonymized with *M. gladiata* by Li (2011). The first true establishment of *M. gladiata* was in 2022 when a heavily infested *F. microcarpa* tree was reported to HDAB (Matsunaga 2022). Immatures are free-living but often produce abundant white flocculence.

###### Genetic resources.

GenBank e.g., MG988795 (COI), MG989108 (cytB) [Taiwan specimen]. A number of other accession numbers for this species can be found on GenBank.

#### Family Liviidae Löw, 1879

**Subfamily Liviinae Löw, 1879**

##### Paurocephala cf. wilderi

Taxon classificationAnimaliaHemipteraLiviidae

Crawford, 1927

2E50B2CA-403B-556C-9310-F5D86C8E5F22

###### First detection/collection in HI.

1925 (Crawford 1927b).

###### Distribution in HI.

Moloka‘i.

###### Material examined.

USA – Hawaiian Islands **·** 1 ♂; Moloka‘i, Kamiloloa; 3200 ft; 20 Dec. 1925; E.H. Bryan leg.; collected on “pokeweed”; BPBM.

###### Host plant in HI.

Uncertain (see remarks).

###### Additional distribution and host plant records.

Endemic to Samoa; adults only, no immatures, but all collections are from *Ficus* (Moraceae) (Mifsud and Burckhardt 2002).

###### Remarks.

This HI record has previously been referred to as *Paurocephala* sp. with native status uncertain (Crawford 1927b; Zimmerman 1948; Percy 2017a). Here we consider *P. cf. wilderi* to be adventive in HI. On re-examination of the single male specimen, we find the species belongs in the *psylloptera*-group *sensu*Mifsud and Burckhardt (2002). Characters, including only a shallow horn on metascutellum, forewing venation including a wide cell cu_1_ and long pterostigma, and reduced number (only 2–3) of stout spurs laterally along the metatibia, would assign this taxon to *P. wilderi*. However, as only a single male specimen was collected in 1925 with no additional specimens recorded for HI, slide preparation of the genitalia (not clearly visible in the dry specimen) would be needed to confirm species designation. Notably, Crawford, who described *P. wilderi* (in Crawford 1927a), did not place the Hawaiian specimen in that taxon when he examined it (Crawford 1927b; Zimmerman 1948). This may be partly due to the obscured genitalia, but also to the brown apical coloration on the forewing that characterizes *P. wilderi* being extremely faint in the HI specimen; additionally, the forewing shape in the HI specimen is wider apically than material from Samoa (as shown in Mifsud and Burckhardt 2002). Although the plant from which the specimen was collected is recorded as “pokeweed”, it is unclear whether this refers to the native pokeweed, *Phytolacca sandwicensis* (Phytolaccaceae) or the introduced species *P. octandra*; both co-occur on Moloka‘i (Wagner et al. 1999). Regardless, *Phytolacca* is unlikely to be a host plant; the *psylloptera*-group are only known from plants in the urticoid Rosales with all adults of *P. wilderi* collected on *Ficus* in Samoa (Mifsud and Burckhardt 2002). As there are no native species of *Ficus* in HI, but many introduced taxa, *Ficus* was the most likely vehicle of introduction into HI. Immature biology unknown.

###### Genetic resources.

None.

#### Family Psyllidae Latreille, 1807

**Subfamily Acizziinae White & Hodkinson, 1985**

##### Acizzia uncatoides

Taxon classificationAnimaliaHemipteraPsyllidae

(Ferris & Klyver, 1932)

918210A9-5A88-5C0C-86A4-BB0E77A376EC

###### First detection/collection in HI.

1966 (Funasaki 1968).

###### Distribution in HI.

Kaua‘i, O‘ahu, Moloka‘i, Lāna‘i, Maui, Hawai‘i.

###### Material examined.

USA – Hawaiian Islands **·** 11 adults; O‘ahu, southern Wai‘anae Mts, Palikea; 21.4098°N, 158.0968°W; 824 m; 08 Apr. 2010; spec. PKSP11274; P.D. Krushelnycky leg.; sweeping; PDK collection **·** 3 adults; O‘ahu, northern Wai‘anae Mtns., Kahanahāiki Valley; 21.5408°N, 158.1940°W; 585 m; 2 May 2009; spec. PKSP3324; P.D. Krushelnycky leg.; ex *Pipturus albidus*; PDK collection **·** 1 ♂, 3 ♀; O‘ahu, northern Ko‘olau Mountains, Pūpūkea; 21. 6419°N, 158. 0031°W; 430 m; 08 Jul. 2014; ex *Metrosideros*; coll. Hi78-14, D.M. Percy leg; DMPC**·** 1 adult; O‘ahu, Ka‘ena Point; 21.5745°N, 158.2745°W; 30 m; 16 Apr. 2015; spec. PKSP47458; P.D. Krushelnycky leg.; sweeping; UHIM**·** 1 adult; O‘ahu, central Wai‘anae Mtns, ‘Ēkahanui; 21.4390°N, 158.0935°W; 658 m; 15 Dec. 2015; spec. COSP3692; C.S. Ogura-Yamada leg.; pitfall trap; PDK collection **·** 8 adults; Maui, Haleakalā NP; Kalahaku; 20.7351°N, 156.2354°W; 2852 m; 20 Jun. 2003; spec. DS04192; P.D. Krushelnycky leg.; ex *Leptecophylla tameiameiae*; PDK collection **·** 2 adults; Maui, Haleakalā NP; Pu‘u o ‘Ili; 20.7466°N, 156.2469°W; 2390 m; 10 Jul. 2003; spec. DS05143; P.D. Krushelnycky leg.; ex *Sophora chrysophylla*; PDK collection **·** 1 adult; Maui, Ko‘olau Forest Reserve, Flume Rd.; 20.48.606°N, 156.14.975°W; 20 Jun. 2006; L. Leblanc leg.; ex methyl eugenol trap; UHIM2015.22504 **·** 3 adults; Maui, Haleakalā NP; 3049 m; 03 Jul. 1968; J.A. Tenorio leg.; UHIM2015.22488 **·** 1 adult; Kaua‘i, Koke‘e; 16 Nov. 1968; J.A. Tenorio leg.; ex light; UHIM2015.22482 **·** 1 adult; Hawai‘i, Mauna Kea, Hale Pōhaku; 19.7599°N, 155.4534°W; 13 Mar. 2015; J. Eiben leg.; ex *Leptecophylla tameiameiae*; UHIM**·** 2 ♀; Moloka‘i, Pālā‘au State Park; 1500’; 2–16 Sept. 1994; W.D. Perreira leg.; yellow sticky board trap; HDAB.

###### Host plants in HI.

*Acacia koa*, *A. melanoxylon*, and *A. confusa* (Fabaceae).

###### Additional distribution and host plant records.

Native to Australia; adventive worldwide, including continental USA; *Acacia* spp., *Albizia* spp. (Fabaceae) (Halbert and Burckhardt 2020; Suh and Choi 2020; Ouvrard 2023).

###### Remarks.

This species was first recorded in HI in 1966 and had spread to most of the islands within a year (Chong and Funasaki 1968). It can be found in abundance on introduced *Acacia* spp. and is a highly invasive pest of endemic *Acacia koa*. Immatures are free-living.

###### Genetic resources.

GenBank MG988634 (COI), MG988909 (cytB) [Hawaiian specimen]. A number of other accession numbers for this species can be found on GenBank.

#### Subfamily Ciriacreminae Enderlein, 1910

##### Euceropsylla orizabensis

Taxon classificationAnimaliaHemipteraPsyllidae

(Crawford, 1914)

1FB5BAAE-423A-5BB1-9113-8DD0E96CD3D3

###### First detection/collection in HI.

2017 (Henry et al. 2018).

###### Distribution in HI.

O‘ahu.

###### Material examined.

None. Material has not been deposited at BPBM or HDAB as indicated in Henry et al. (2018) but may be deposited at USNM.

###### Host plant in HI.

*Pithecellobium dulce* (Fabaceae).

###### Additional distribution and host plant records.

Native to Central America; *Inga* spp. (Fabaceae) (Brown and Hodkinson 1988).

###### Remarks.

This is an unusual instance where an adventive species recorded for HI, as well as the host plant association (Henry et al. 2018), are not otherwise known outside the native range. As several *Euceropsylla* species groups are recognized that include highly similar species, and no type material was examined in the determination of HI specimens, presence of this species should be confirmed with additional collections. Immatures are free-living.

###### Genetic resources.

None.

##### Heteropsylla cubana

Taxon classificationAnimaliaHemipteraPsyllidae

Crawford, 1914

03BA5A6F-968C-511A-B6BA-2301D85BBF8A

###### First detection/collection in HI.

1984 (Nakahara 1986a; Beardsley and Uchida 1990; see Remarks).

###### Distribution in HI.

Kaua‘i, O‘ahu, Moloka‘i, Lāna‘i, Maui, Hawai‘i.

###### Material examined.

USA – Hawaiian Islands **·** 1 ♂, 2 ♀; Kaua’i, Waimea; 31 Mar. 1995; R. Oyama leg.; ex corn; HDAB**·** 1 ♂, 2 ♀; Lāna‘i, Lāna‘i City; 28 Jun. 1984; W. Nagamine leg.; ex *Leucaena leucocephala*; HDAB**·** ~20 adults; Hawai‘i, south of Waimea, Hwy 190; 20.0108°N, 155.6762°W; 820 m; 17 Jul. 2013; D.M. Percy leg.; ex *Leucaena leucocephala*; coll. Hi36-13; DMPC**·** 6 ♂, 10 ♀, 2 immatures; O‘ahu, Ka‘ena Point; 21.5732°N, 158.2749°W; 26 m; 16 Apr. 2015; spec. PKSP44454; P.D. Krushelnycky leg.; beating; PDK collection **·** 1 ♂, 1 ♀; O‘ahu, southern Wai‘anae Mtns, Palikea; 21.4142°N, 158.0994°W; 900 m; 16 Mar. 2011; spec. PKSP16277; P.D. Krushelnycky leg.; sweeping; PDK collection **·** 1 ♀; O‘ahu, northern Wai‘anae Mtns., Kahanahāiki Valley; 21.5406°N, 158.1940°W; 588 m; 14 Dec. 2009; spec. PKSP10937; P.D. Krushelnycky leg.; ex *Pipturus albidus*; PDK collection **·** 1 ♂; O‘ahu, northern Wai‘anae Mtns., Pahole Natural Area Reserve; 21.5408°N, 158.1882°W; 509 m; 15 Dec. 2009; spec. PKSP7028; P.D. Krushelnycky leg.; pitfall trap; PDK collection.

###### Host plants in HI.

*Leucaena leucocephala*, *Samanea saman* (Fabaceae).

###### Additional distribution and host plant records.

Native to Central and South America but adventive and now widely distributed in the Pacific and other tropical and subtropical regions; adventive on native *Schleinitzia insularum* (Fabaceae) in the South Pacific (Muddiman et al. 1992; Claridge et al. 2014; Halbert and Burckhardt 2020; Martoni et al. 2025).

###### Remarks.

Recorded for HI by Beardsley and Uchida (1990) and also previously reported as *Heteropsylla sp. nr. fusca* (Crawford) and *H.* sp. poss. *incisa* (Šulc) (Nakahara 1986a, 1986b, 1988; Nagamine et al. 1992). Beardsley and Uchida (1990) reported four parasitoid species raised from immatures. Immatures are free-living.

###### Genetic resources.

GenBank PX507510 (COI), PX527535 (cytB) (Table 3), SRR1821923 (transcriptome) [Hawaiian specimens]. A number of other accession numbers for this species can be found on GenBank.

##### Heteropsylla fusca

Taxon classificationAnimaliaHemipteraPsyllidae

Crawford, 1914

6411BEAD-37E4-5F7D-8587-390F99831668

###### First detection/collection in HI.

1986 (Nakahara 1988).

###### Distribution in HI.

O‘ahu.

###### Material examined.

USA – Hawaiian Islands **·** 1 ♂, 1 ♀; O‘ahu, St. Louis Hts.; 4 Jan. 1986; ex *Acacia* [=*Vachellia*] farnesiana; HDAB.

###### Host plant in HI.

*Vachellia farnesiana* (Fabaceae).

###### Additional distribution and host plant records.

North, Central and South America; *Acacia* spp., *Haemaloxylon campechianum*, *H. brasiletto* (Fabaceae) (Muddiman et al. 1992; Halbert and Burckhardt 2020; Horton et al. 2021; Ouvrard 2023).

###### Remarks.

First published for HI in 1988 (Nakahara 1988). Immatures are free-living.

###### Genetic resources.

None.

##### Heteropsylla huasachae

Taxon classificationAnimaliaHemipteraPsyllidae

Caldwell, 1941

C82C93CC-3745-5814-913D-E1AC656B284E

###### First detection/collection in HI.

1975 (Beardsley 1977; Nagamine et al. 1992; see Remarks).

###### Distribution in HI.

Kaua‘i, O‘ahu.

###### Material examined.

USA – Hawaiian Islands **·** 1 ♂; O‘ahu, Ewa; 24 Mar. 1977; J.W. Beardsley leg.; ex *Desmanthus virgatus*; UHIM2015.22520 **·** 1 ♀; O‘ahu, Honolulu International Airport; 08 Feb. 1977; J.W. Beardsley leg.; ex light trap; UHIM2015.22528 **·** 7 ♂; ‘Ewa; 9 Apr. 1985; K. Murai leg.; ex *Desmanthus*; coll. Oa 85-77; HDAB.

###### Host plants in HI.

*Acacia koa*, *Desmanthus virgatus*, *Samanea saman* (Fabaceae).

###### Additional distribution and host plant records.

Widespread in Central America and Caribbean islands, occurs as adventive in South and North America; *Albizia* spp. (Fabaceae) (Brown and Hodkinson 1988; Muddiman et al. 1992; Ouvrard 2023).

###### Remarks.

Previously reported as *Heteropsylla nr. fusca*, *H.* sp. poss. *mimosae*, *H.* sp. prob. *mimosae*, *H.* sp. 2, *H.* sp. 3 (Beardsley 1977; Nakahara 1980, 1986b, 1988; Nagamine et al. 1992). Lab tests by Nagamine et al. (1992) showed *Acacia koa* to be a weak, or sub-optimal, host plant. Immatures are free-living.

###### Genetic resources.

None.

##### Heteropsylla texana

Taxon classificationAnimaliaHemipteraPsyllidae

Crawford, 1914

2BC19E82-93C7-5FEE-93CF-CE44CDDDD067

###### First detection/collection in HI.

1999 (new state record; see Remarks).

###### Distribution in HI.

Kaua‘i, O‘ahu.

###### Material examined.

USA – Hawaiian Islands **·** 1 ♂, 1 ♀; Kaua‘i, Nohili Dunes, Barking Sands, Pacific Missile Range Facility; 22.0621°N, 159.7833°W; 24 Mar. 2021; yellow pan traps placed below *Prosopis* [=*Neltuma*] pallida; K. Magnacca & J. Preble leg.; coll. 2022-165; HDAB**·** 2 ♂; O‘ahu, Sand Island; 21.3094°N, 57.8871°W; 30 Jun. 2022; M. Ramadan leg.; ex *Neltuma pallida*; coll 2022-176; HDAB.

###### Host plant in HI.

*Neltuma pallida* (Fabaceae).

###### Additional distribution and host plant records.

Widespread in southern continental USA and Mexico; several species of *Neltuma* (=*Prosopis*) and also *Pithecellobium* sp. (Fabaceae) (Muddiman et al. 1992; Percy et al. 2012; Ouvrard 2023).

###### Remarks.

In 2022, two individuals (1 ♂, 1 ♀) were collected in a yellow pan trap placed beneath *Neltuma pallida* trees at Barking Sands Beach, Pacific Missile Range Facility, Kaua‘i. Identification was confirmed by Cheryle O’Donnell (USNM). Initially thought to be a new state record until an unpublished HDOA report came to light with a citing of *H. texana* collected at Ke‘ehi Lagoon, O‘ahu in 1999. The report stated “Surveys disclose *Prosopis* [=*Neltuma*] (kiawe), *Pithecellobium* (opiuma), *Chrysopsis*, *Tamarix*, and *Acacia* (koa) as hosts.” However, no voucher specimens could be located to verify this, and it seems that the record was never publicly published. A survey of the Ke‘ehi Lagoon in 2022 confirmed establishment of *H. texana* on *N. pallida*. For this reason, we report *Heteropsylla texana* as a new state record here. Immatures are free-living.

###### Genetic resources.

GenBank e.g., MG988760 (COI), MG989069 (cytB) [California specimen]. A number of other accession numbers for this species can be found on GenBank.

#### Subfamily Diaphorininae Vondráček, 1951

##### Diaphorina citri

Taxon classificationAnimaliaHemipteraPsyllidae

Kuwayama, 1908

B5F45327-EE24-586A-963A-FA11DB49C80E

###### First detection/collection in HI.

2006 (Conant et al. 2006).

###### Distribution in HI.

Kaua‘i, O‘ahu, Moloka‘i, Lāna‘i, Maui, Hawai‘i.

###### Material examined.

USA – Hawaiian Islands **·** 8 ♂, 8 ♀; O‘ahu, Pāwa‘a, [HDOA]; 16 Aug. 2007; B. Kumashiro leg.; ex *Murraya paniculata*; coll. 2007-099; HDAB**·** 3 ♀; O‘ahu, Waipahu, Kunia; 4 Nov. 2008; J.N. Matsunaga leg.; ex *Citrus sinensis*; coll. 2008-183; HDAB

###### Host plant in HI.

*Citrus* spp. and *Murraya paniculata* are major hosts (Rutaceae).

###### Additional distribution and host plant records.

Native to southeast Asia; adventive worldwide; *Citrus* spp., *Murraya* spp., and closely related genera (Rutaceae) (Percy et al. 2012; Halbert and Burckhardt 2020; Ouvrard 2023).

###### Remarks.

This species was first detected on the island of Hawai‘i, and rapidly spread to all the other main islands (Conant et al. 2006; Matsunaga 2014). Oddly, it had been listed by Nishida (2002) but without any island distribution, and we can find no earlier records. Considered a serious pest of *Citrus*, particularly as the vector of citrus greening disease (huanglongbing) (Ghosh et al. 2022; Higgins et al. 2022). Currently, the disease agents, *Candidatus Liberibacter* spp., are not known to be present in HI. Two known parasitoids of this species, the eulophid *Tamarixia cf. radiata* (Waterston) and encyrtid *Diaphorencyrtus aligarhensis* (Shafee, Alam, & Agarwal), also arrived in HI as fortuitous biocontrol agents (Matsunaga 2014). Immatures are free-living.

###### Genetic resources.

GenBank e.g., MG988723 (COI), MF426268 (complete mitochondrion) [Taiwan specimen]. A number of other accession numbers for this species can be found on GenBank.

#### Subfamily Psyllinae Latreille, 1807

##### Cacopsylla tobirae

Taxon classificationAnimaliaHemipteraPsyllidae

(Miyatake, 1964)

64272AB3-FDD6-5D03-A29F-17483B56844D

###### First detection/collection in HI.

2013 (Matsunaga et al. 2019).

###### Distribution in HI.

O‘ahu.

###### Material examined.

USA – Hawaiian Islands **·** 7 ♂, 7 ♀, 10 immatures; O‘ahu, Mānoa, UH; 8 Jul. 2013; S. Nelson leg.; ex *Pittosporum tobira*; coll. 2013-211; HDAB, USNM**·** 1 ♂, 1 ♀; O‘ahu, Pāwa‘a; 9 Jul. 2013; M. Tauyan leg.; ex *Pittosporum tobira*; coll. 2013-208; HDAB**·** 2 ♂, 20 immatures; O‘ahu, Mānoa, UH, East West Center; 31 May 2017; J.N. Matsunaga, K. Wong leg.; ex *Pittosporum tobira*; coll. 2017-126; HDAB.

###### Host plant in HI.

*Pittosporum tobira* (Pittosporaceae).

###### Additional distribution and host plant records.

Native to Asia (Japan, South Korea, Taiwan); adventive in California, North and South Carolina (Miyatake 1964; Percy et al. 2012; Bertone 2016), intercepted but not considered established in Florida (Halbert and Burckhardt 2020). The host plant in HI is the only recorded host.

###### Remarks.

First collected in 2013, heavily infesting *Pittosporum tobira* from Pāwa‘a through Mānoa, Honolulu, O‘ahu areas (Matsunaga et al. 2019). Possibly now under control by parasitoids, although active removal of many of the host plants may also have impacted survival. Immatures are free-living.

###### Genetic resources.

GenBank e.g., OR074490 [Florida specimen]. A number of other accession numbers for this species can be found on GenBank.

#### Family Triozidae Löw, 1879

##### Heterotrioza chenopodii

Taxon classificationAnimaliaHemipteraTriozidae

(Reuter, 1876)

6BD4AB38-4B69-5ECD-BEEC-F6DF03ACB688

###### First detection/collection in HI.

2010 (Percy et al. 2020).

###### Distribution in HI.

O‘ahu.

###### Material examined.

USA – Hawaiian Islands **·** 1 ♂, 2 ♀; O‘ahu, Ka‘ena Point; 21.5726°N, 158.2797°W; 9–19 m; 16 Apr. 2015; spec. PKSP46529, PKSP45882, PKSP44470; P.D. Krushelnycky leg.; ex various Chenopodiaceae including *Atriplex suberecta*; coll. T4-PDK980-15; BPBM, DMPC, HDAB, UHIM.

###### Host plants in HI.

A likely host plant is *Atriplex suberecta*, and possibly *A. semibaccata* and *Chenopodium oahuense* (Amaranthaceae).

###### Additional distribution and host plant records.

Widespread Palearctic species known from North America since 1980s; in addition to *Atriplex* spp. and *Chenopodium* spp. other host genera recorded include *Beta* and *Spinacia* (Amaranthaceae) (Horton et al. 2018; Duarte et al. 2025).

###### Remarks.

First published records for HI in 2020 (Percy et al. 2020). Immatures are free-living.

###### Genetic resources.

GenBank MT162454–MT162455 (COI), MT176418–MT176419 (cytB) [Hawaiian specimens]. A number of other accession numbers for this species can be found on GenBank.

##### Leptynoptera sulfurea

Taxon classificationAnimaliaHemipteraTriozidae

Crawford, 1919

2CE791C9-825B-5791-98D5-9C92CB61A884

###### First detection/collection in HI.

1977 (Beardsley 1981).

###### Distribution in HI.

Kaua‘i, O‘ahu, Moloka‘i, Maui, Hawai‘i.

###### Material examined.

USA – Hawaiian Islands **·** 1 ♀; O‘ahu, Hickam AFB; 23 Nov. 1977; J.W. Beardsley leg.; ex *Calophyllum*; UHIM2015.22572 **·** 2 ♂, 2 ♀, 20 immatures; same data as previous; HDAB**·** 1 ♂, 3 ♀, 10 immatures; Kāne‘ohe, Ho‘omaluhia Botanical Garden; 24 Jul. 2013; W. Nagamine leg.; ex *Calophyllum brasiliense*; coll. 2013-225; HDAB**·** 6 ♂, 4 ♀; Maui, Lahaina, Lahainaluna School; 30 Mar. 1993; R. Sher leg.; ex *Calophyllum inophyllum*; coll. 1993-109; HDAB**·** 5 ♂, 5 ♀; Kaua‘i, Līhu‘e Airport; 25 Mar. 1993; C. Campbell leg.; ex *Calophyllum inophyllum*; coll. 1993-107; HDAB**·** 1 ♀; Moloka‘i, Halawa Valley; 61 m; 03 Mar. 1995; W.D. Perreira leg.; yellow sticky trap; UHIM2015.22578.

###### Host plants in HI.

*Calophyllum inophyllum*, and possibly *C. brasiliense* (Calophyllaceae).

###### Additional distribution and host plant records.

Broadly distributed in Asia-Pacific region; *Calophyllum* spp. (Calophyllaceae) (Martin and Hollis 1992; Martoni and Brown 2018).

###### Remarks.

First reported for HI by Beardsley (1981). Although this species is widespread, and in many Pacific regions, such as French Polynesia and the Cook Islands, it is considered likely native – the Hawaiian occurrence is considered adventive due to the absence of HI records prior to 1970s. Immatures are free-living.

###### Genetic resources.

GenBank e.g., KY293709 (COI), MG989229 (complete mitochondrion) [New Caledonia specimen]. A number of other accession numbers for this species can be found on GenBank.

### *Australopsylla* – additional resources and diagnostic key

**Figure 15.**
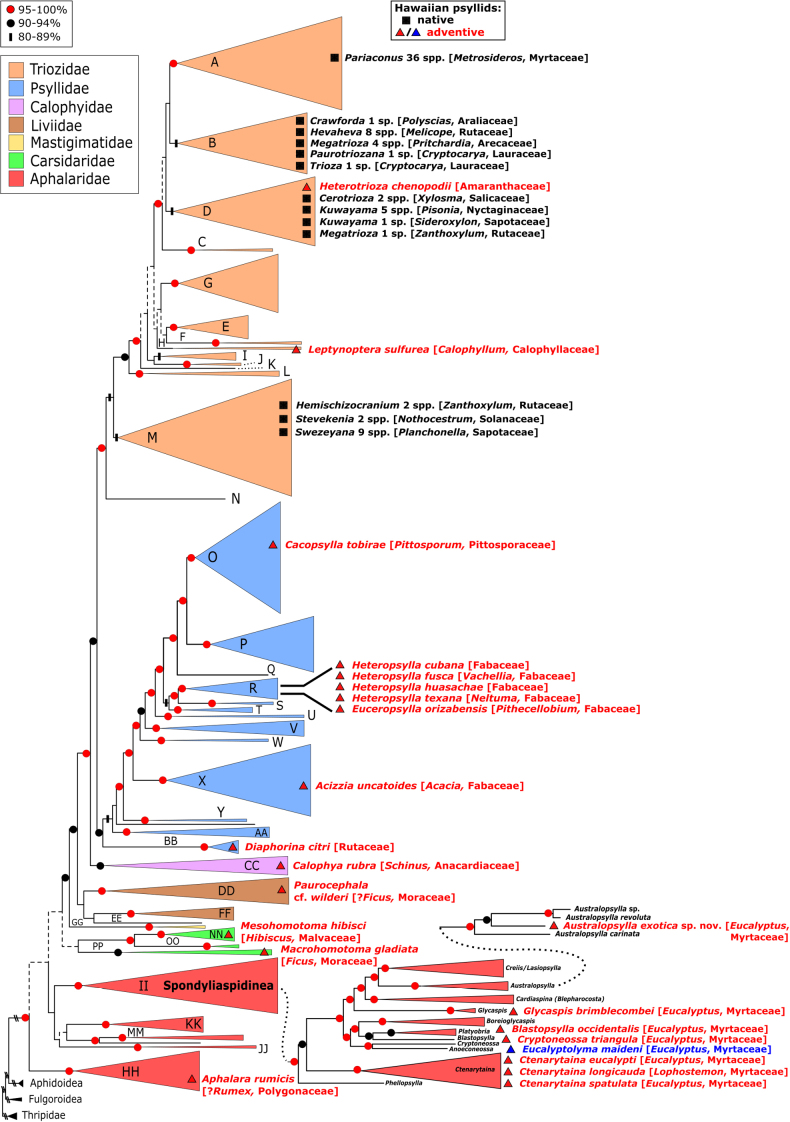
Systematic placement of the Hawaiian Island psyllid fauna (native taxa represented by black boxes, adventive taxa represented by red or blue triangles) based on previously published data (Percy et al. 2018) and new sequence data from this study (Table 3). The tree represents the mitogenome analysis of Percy et al. (2018) (all genera treated here that are included using original or newly generated molecular data are in black or red; genera without molecular data in blue), with updated classification according to Burckhardt et al. (2021). Placement of nine taxa not included in the original mitogenome analysis (Percy et al. 2018) are determined using a maximum likelihood backbone constraint analysis (see Methods). The more detailed clade placement within subfamily Spondyliaspidinae shows the placement of *Australopsylla*, and *A. exotica* Matsunaga & Percy, sp. nov. relative to the other two described *Australopsylla* species and *Australopsylla* sp. *sensu* Tuthill and Taylor (1955: 237). *Blepharocosta* was recovered within *Cardiaspina* but with support < 80%. *Eucalyptolyma*, for which we do not have molecular data, is positioned here close to *Anoeconeossa* (according to Taylor 1987a; Martoni et al. 2018). Host plant genera and families are given for HI records if known, otherwise extrapolated from host data elsewhere.

Here we provide additional data for the newly described species, *A. exotica* sp. nov., with reference to related taxa. Genetic data generated for this study indicates the placement of the newly described species relative to described taxa (Fig. 15) as well as to a fourth undescribed species that is likely the same as “*Australopsylla* sp.” figured in Tuthill and Taylor (1955: 237) and Morgan (1984: 33); this species is similar to *A. exotica* sp. nov. in the apical pattern of the female forewing partially covering cell cu_1_, but lacks the distinct spots found in the forewing pattern of *A. exotica*. Another species from South Australia, mislabelled as “*Australopsylla marmorata*” in Morgan (1984: 66), is another undescribed species which also has the apex of the female forewing notably darker, in this case as a solid block that entirely covers cell cu_1_ (Morgan 1984: plate 19(3)).

**Figure 16.**
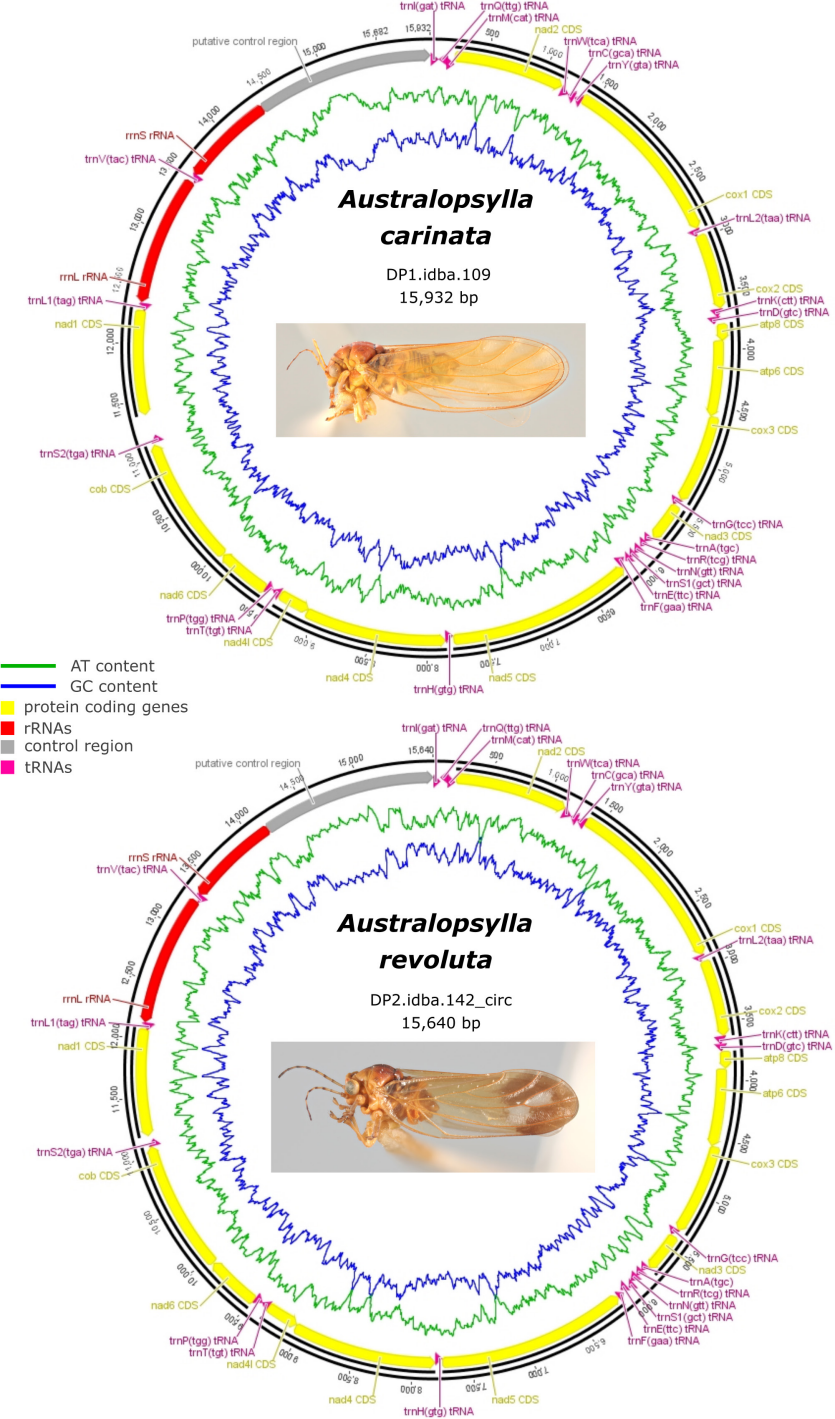
Graphical representation of mitogenome annotations for *Australopsylla carinata* and *A. revoluta*. Images of females of the respective *Australopsylla* species (ANIC specimens) taken by Olivia Evangelista de Souza.

**Genetic resources**. Short mitochondrial sequences of *A. carinata* and *A. revoluta* are on GenBank, but here we provide the complete annotated mitochondrial genomes (Fig. 16) for *A. carinata* and *A. revoluta* (the latter is the type species of the genus) to contribute to the clarification of *Australopsylla* systematics; these two taxa were included in the mitogenome analysis of Percy et al. (2018), but the annotated mitogenomes have not previously been published. GenBank: *A. carinata* (as “DP1.idba.109 *Australopsylla* sp.” in Percy et al. 2018), MG988647 (COI), MG988932 (cytB), PX549493 (complete mitochondrion); *A. revoluta* (as “DP2.idba.142_circ *Australopsylla* sp.” in Percy et al. 2018), MG988646, MT375258 (COI), MG988933 (cytB), PX549492 (complete mitochondrion); *A.* sp. *sensu* Tuthill and Taylor (1955: 237) PX507504 (COI), PX527529 (cytB) (Table 3). A number of other accession numbers for *A. revoluta* can be found on GenBank.

**Material examined for *Australopsylla carinata***. Australia – ACT **·** 1 ♀; Namadgi; 02 Nov. 2001; coll. Aust01-01; D.M. Percy leg.; ex *Eucalyptus dives*; DMPC. Paratype and other specimens were examined from images only: Australia – NSW **·** Paratype 1 ♀; Sydney, Mos[man?] Bay; 20 Aug. 1893; W.W. Froggatt leg.; ANIC. Australia – ACT **·** several adults; ACT, Mnt. Ginini; 03 Mar. 1953; Common & Riek leg.; ex *Eucalyptus niphophila* [=*Eucalyptus pauciflora* subsp. niphophila]; ANIC.

**Material examined for *Australopsylla revoluta***. Australia – ACT **·** 9 ♂, 4 ♀; Tharwa; 18 Aug. 2001; coll. AuS25-01; D.M. Percy leg.; ex *Eucalyptus viminalis*; DMPC. Paratype and other specimens were examined from images only: Australia – VIC **·** Paratypes 1 ♂, 2 ♀; 20 Aug. 1893; W.W. Froggatt leg. ANIC**·** lerps; 10 Jun. 1893; W.W. Froggatt leg.; ex *Eucalyptus gracilis*; ANIC. Australia – ACT **·** 6 ♀; Ainslie; 29 Sep. 1951; K.L. Taylor leg.; ex *Eucalyptus macarthurii*; ANIC.

**Material examined for *Australopsylla* sp. *sensu* Tuthill and Taylor (1955: 237)**. Australia – ACT **·** 1 ♀; Canberra; 26 Nov. 2001; coll. AuAusSpF01; D.M. Percy leg.; ex *Eucalyptus leucoxylon*; DMPC.

### Key to adults of *Australopsylla* (described species)

**Table:** 

1	Forewing membrane of male and female not dimorphic, concolorous, ochreous throughout (i.e., lacking distinct darker apical pattern of bands and/or spots in females) (Fig. 7A); anal ring entire and not laterally expanded outwards and upwards on anterior humps of female proctiger (Fig. 10B)	***A. carinata* (Froggatt, 1900)** [Australia]
–	Forewing membrane of male and female dimorphic, uniformly ochreous to suffused darker towards apex in males, females with distinctly darker brown pattern of bands and/or spots in the apical portion (Fig. 7B–D); anal ring not entire (having a short discontinuity in mid-anterior section) and laterally expanded outwards and upwards on anterior humps of female proctiger (Figs 9G–I, 10D)	**2**
2	Larger species, WL > 3 mm (~ 3.1–4.2 mm, Fig. 7B, Table 2); female forewing membrane, when compared to male, with distinctly darker brown band around the apical margin, and broadly along veins M_3+4_ and Cu_1a_ and entirely covering apical half of cell m_2_, remainder of membrane fuscous except a clear window in anterior part of cell m_1_ into cell r_2_ (Fig. 7B); antenna longer, AL:HW > 1.4 (Fig. 8D, Table 2); male terminalia (in lateral view) with subgenital plate ventral margin more or less straight, paramere anterior margin medially constricted, distal aedeagus segment with mid-dorsal projection (Fig. 11I)	***A. revoluta* (Froggatt, 1900)** [Australia]
–	Smaller species, WL ≤ 3 mm (~ 2.3–3.0 mm, Fig. 7C, D, Table 2); female forewing membrane, when compared to male, with distinctly darker brown apical portion consisting of spots and a dark brown band around the apical margin (Fig. 7C); antenna shorter, AL:HW ≤ 1.4 (Fig. 8F, Table 2); male terminalia (in lateral view) with subgenital plate ventral margin rounded, paramere anterior margin not medially constricted (at most slight constriction towards base), distal aedeagus segment without mid-dorsal projection (Fig. 11E)	***A. exotica* Matsunaga & Percy, sp. nov**. [Australia and HI]

## Discussion

With the records newly reported here for psyllids, approximately 470 species of Sternorrhyncha (aphids, psyllids, scale insects, and whiteflies) are recorded for the Hawaiian Islands, and a little >77% are considered adventive; the native Sternorrhyncha species now account for just under 23%. This continuing shift towards the overdominance of adventive species in the Hawaiian fauna is exacerbated by the decline of native plant populations that host native sternorrhynchan insects (e.g., Percy 2017b; Percy et al. 2024), with recent reports indicating >70% of the native vascular flora is considered threatened (Wood et al. 2019; Rønsted et al. 2022).

The native Hawaiian Sternorrhyncha fauna comprises just two superfamilies: psyllids (Psylloidea) and scale insects (Coccoidea, now usually referred to as Coccomorpha), with the number of described native psyllids (73 spp. in 11 genera) more than double the number of described native scale insects (34 spp. in 14 genera). The native species in both groups are all Hawaiian endemics (Hembry et al. 2021). In addition, the ratio of native to adventive taxa in these two superfamilies is markedly different: <16% of the 215 Coccomorpha species, but 76% of the 96 Psylloidea species are native. In comparison with the Canary Islands (CI), an archipelago of comparable size and complexity, although much less geographically isolated, there is similar disparity in endemism between the two superfamilies: CI endemism for Coccomorpha is 19% and for Psylloidea it is 66% (Bastin et al. 2024). Similarly, comparable numbers are found for the native versus adventive psyllid taxa (native/adventive: HI 76%/24%, CI 80%/20%). Notably, and this directly relates to the difference in geographical isolation (CI distance to North Africa ~100–400 km, whereas HI >3000 km to the nearest continent) are the number of colonization events: the native Hawaiian psyllid fauna results from as few as eight colonization events (Hembry et al. 2021), whereas the native Canarian (plus Madeira) psyllid fauna results from an estimated 26 colonization events (Bastin et al. 2024). Surprisingly, there are no native lineages of aphids (Aphidomorpha) or whiteflies (Aleyrodoidea) in the Hawaiian Islands, whereas in the Canary Islands 24% of Aleyrodoidea are endemic, but <1% of Aphidomorpha are endemic.

It is striking that all the native Hawaiian psyllids are in the family Triozidae, and all but two of the introduced psyllid species are in other families. This allows for relatively easy recognition of adventive versus native taxa, as most of the adventive species will not have the distinct trifurcating forewing venation characteristic of Triozidae. Of the two adventive Triozidae species, only one, *Heterotrioza chenopodii* (on O‘ahu), could potentially be confused with native taxa (e.g., *Pariaconus ohiacola* also on O‘ahu) based on forewing morphology, although both the overall forewing shape and particularly the height of cell cu_1_ are diagnostic in this instance (Percy et al. 2020). Moreover, these two species are very unlikely to occur in the same area because *H. chenopodii* is found in semi-arid, low elevation vegetation, and *P. ohiacola* is found in native ohia shrubland at higher elevations (Percy 2017a; Percy et al. 2020). The other adventive triozid (on O‘ahu and other islands), *Leptynoptera sulfurea*, has a unique forewing morphology lacking a cubital cell (cu_1_), a characteristic not shared with any of the native genera. The phylogenetic overview presented here serves to emphasize the systematic clumping of native versus adventive groups in the Hawaiian Islands, and in particular, the high number of adventive species in Aphalaridae subfamily Spondyliaspidinae, which includes all the Myrtaceae-feeding taxa with Australasian origins.

Several of the eucalypt-feeding species found in the Hawaiian Islands are considered pests elsewhere, but they do not appear to be having a seriously adverse effect on eucalypts in the Hawaiian Islands. Considerable numbers of several unrecorded species of the parasitoid wasp genus *Psyllaephagus* (Hymenoptera: Encyrtidae) are usually taken when sweeping *Corymbia citriodora* and other eucalypts in HI, and these parasitoids may be preventing eucalypt psyllids from establishing large populations. However, none appear to be *P. parvus* Riek or *P. perplexus* Riek, which have been recorded attacking *Eucalyptolyma maideni* and *Cryptoneossa triangula* respectively in California (Eatough Jones et al. 2011). Additional parasitoid species, *P. bliteus* Riek, *P. pilosus* Noyes, and *P. yaseeni* Noyes, are already known to be established in HI and attacking *Glycaspis brimblecombei*, *Ctenarytaina eucalypti*, and *Heteropsylla cubana* respectively (Nishida 2002; Matsunaga et al. 2019).

Checklists, like this one, provide only a snapshot of the adventive fauna as rates of insect introductions around the globe continue to increase (Seebens et al. 2021). It is probable that there will be both an increase in number and type of introduced psyllid species, for instance, occurrences of species not previously considered typical adventives (e.g., *Australopsylla exotica* and *Euceropsylla orizabensis*). Moreover, increasing imports and cultivation of a diversity of exotic plant species will make the permanent establishment of adventives more likely. For example, two species recorded historically for HI, *Aphalara rumicis* in 1947 and *Paurocephala cf. wilderi* in 1925, did not become permanently established, possibly because suitable plants were absent or insufficient, but if arriving today, these and other species are more likely to established because of the greater diversity of introduced and established invasive plants. In this example, these two species were collected (each as a single individual) by chance, and thus, by extrapolation, other arrivals may have gone unrecorded.

Other taxa not yet recorded for the Hawaiian Islands, but in some cases already widely established as adventives elsewhere, are of particular concern, such as additional *Acizzia* and *Heteropsylla* species, the Africa *Citrus* psyllid (*Trioza erytreae* (Del Guercio)), and several *Bactericera* spp. including the widespread potato/tomato psyllid (*Bactericera cockerelli* (Šulc)) (Muddiman et al. 1992; Suwandharathne et al. 2023; Taylor et al. 2023; Bertinelli et al. 2024; Pérez-Otero et al. 2024). Additional *Eucalyptus*-feeding and other Myrtaceae-feeding species originating in Australasia should be expected based on the recent increase of this group reported here. There are also psyllids found on tropical fruit crops that HI should be vigilant for, such as durian (e.g., *Allocarsidara malayensis* (Crawford)) and additional *Ficus*-feeding psyllids (e.g., *Mycopsylla fici* (Tryon)); some *Ficus* psyllids (e.g., *Macrohomotoma gladiata*, already established in HI) are *Candidatus Liberibacter* vectors, though thus far not considered pathogenic (Lin et al. 2022). There are also South American potato and pepper psyllids (*Russelliana solanicola* Tuthill and *Russelliana capsici* Burckhardt) that are known to be spreading beyond their native range and vectoring potential pathogens (Syfert et al. 2017; Kwak et al. 2021; Kuhn et al. 2023). Perhaps most critically, the arrival of more *Diaphorina citri* individuals – an Asian species and potential plant disease vector that is already established in HI and elsewhere, including continental USA – that are actively harbouring and can vector the causal agent of citrus greening disease (huanglongbing), should be an ongoing concern. This disease has not yet been detected in HI but has the potential to cause immense damage to Hawaiian fruit production (Hall et al. 2013; Britt et al. 2020; Leong et al. 2022). Additionally, despite much scientific focus on this pathogen in recent years, there remain challenges to detection (initial infection may be asymptomatic), as well as for prevention and mitigation (Arredondo Valdés et al. 2016; Kennedy et al. 2023; Volpe et al. 2024). For this as well as other potential threats, vigilance, ongoing surveys, and screening using molecular assays (e.g., as reviewed in Patel et al. 2022) are the best defenses.

## Supplementary Material

XML Treatment for Aphalara rumicis

XML Treatment for Australopsylla

XML Treatment for Australopsylla exotica

XML Treatment for Blastopsylla occidentalis

XML Treatment for Cryptoneossa triangula

XML Treatment for Ctenarytaina eucalypti

XML Treatment for Ctenarytaina longicauda

XML Treatment for Ctenarytaina spatulata

XML Treatment for Eucalyptolyma maideni

XML Treatment for Glycaspis brimblecombei

XML Treatment for Calophya rubra

XML Treatment for Mesohomotoma hibisci

XML Treatment for Macrohomotoma gladiata

XML Treatment for Paurocephala cf. wilderi

XML Treatment for Acizzia uncatoides

XML Treatment for Euceropsylla orizabensis

XML Treatment for Heteropsylla cubana

XML Treatment for Heteropsylla fusca

XML Treatment for Heteropsylla huasachae

XML Treatment for Heteropsylla texana

XML Treatment for Diaphorina citri

XML Treatment for Cacopsylla tobirae

XML Treatment for Heterotrioza chenopodii

XML Treatment for Leptynoptera sulfurea

## Additional information

### Artificial Intelligence (AI) use

The authors accept full responsibility for the content of the manuscript, including the disclosure of any use of AI.

No AI tools were used in the preparation of this manuscript.

### Author ORCIDs

J.N. Matsunaga https://orcid.org/0009-0004-4148-7334

K.N. Magnacca https://orcid.org/0000-0003-3803-7506

P.D. Krushelnycky https://orcid.org/0009-0003-7569-5986

D.M. Percy https://orcid.org/0000-0002-0468-2892

### Data availability

All sequences generated for this study are deposited in GenBank (accession numbers provided under Genetic Resources and in Table 3). Sequence alignment files are available from the corresponding author (DMP) on request. All other data is contained in this article.
